# Assessment of Juniper Ash Elemental Composition for Potential Use in a Traditional Indigenous Dietary Pattern

**DOI:** 10.3390/nu18020260

**Published:** 2026-01-14

**Authors:** Julie M. Hess, Madeline E. Comeau, Derek D. Bussan, Kyra Schwartz, Claudia PromSchmidt

**Affiliations:** 1 US Department of Agriculture, Agriculture Research Service, Grand Forks Human Nutrition Research Center, 2420 2nd Ave N, Grand Forks, ND 58203, USA; 2 Department of Nutrition & Dietetics, University of North Dakota, Grand Forks, ND 58202, USA; 3 School of Medicine and Health Sciences, University of North Dakota, Grand Forks, ND 58202, USA

**Keywords:** juniper ash, indigenous, nutrient, calcium, minerals, lead

## Abstract

**Background/Objectives:** Ash made from juniper trees and added to cornmeal-based dishes may have provided calcium (Ca) to traditional Indigenous diets. Few studies have quantified the mineral content of juniper ash, including its Ca content. The objective of this study was to determine whether juniper ash could serve as a safe source of non-dairy Ca in an intervention study. **Methods:** Branches from two varieties of Juniper (Rocky Mountain Juniper, or *Juniperus scopulorum* and Eastern Red Cedar, or *Juniperus virginiana)* were harvested and burned to ash in a laboratory setting. Juniper ash from the southwestern U.S. available for retail purchase was used for comparison. All samples were tested for content of 10 nutritive elements (Ca, copper, iron, potassium, magnesium, manganese, sodium, phosphorus, selenium, and zinc) and 20 potentially toxic elements (silver, aluminum, arsenic, barium, beryllium, cadmium, cobalt, chromium, mercury, lithium, molybdenum, nickel, lead, antimony, tin, strontium, thallium, uranium, and vanadium) as well as n = 576 pesticide residues. **Results:** All samples contained both nutritive and potentially toxic elements. Each teaspoon of ash contained an average of 445 ± 141 mg Ca. However, the samples also contained lead in amounts ranging from 1.09 ppm to 15 ppm. **Conclusions:** Information on the nutritive and potentially toxic elemental content of juniper ash and how it may interact within a food matrix is insufficient to determine its safety as a Ca source. Further investigation is needed on the bioavailability of calcium oxide and its interaction with other dietary components to clarify the potential role of juniper ash in contemporary food patterns.

## 1. Introduction

Several federally recognized tribal communities experience greater rates of health disparities than surrounding areas, which may be due in part to a forced transition away from traditional diets [1,2]. The United States Department of Agriculture (USDA)- Agricultural Research Service (ARS) Grand Forks Human Nutrition Research Center (GFHNRC) is located within close geographic proximity to several federally recognized tribal communities. As part of the GFHNRC mission to improve the understanding of the impact of food choice on health, an intervention study assessing the impact of a healthy traditional Indigenous diet (TID) on several health outcomes among Indigenous people living in the Grand Forks, ND area was planned [3]. The TID includes foods consumed within the Great Plains region prior to the year 1851, when the Treaty of Fort Laramie was signed, establishing boundaries between Indian tribes of the Northern Great Plains [3,4]. The TID was also developed to provide a similar nutrient profile to that provided by the Healthy-U.S.-Style Dietary Pattern in the 2020–2025 Dietary Guidelines for Americans (DGA) [5].

Several commonly consumed ingredients recommended in the DGA, including dairy foods, were not consumed as part of traditional Indigenous dietary patterns in the Northern Great Plains. Historic records indicate that cattle were brought to the United States from Europe, but cattle breeds were not utilized specifically for dairy in North America until the late 1800s [6]. Before European colonization of North America, utilizing cows for milk consumption was confined to Central and Northern Europe, the Middle East, sub-Saharan Africa, Central Asia, and the Indian subcontinent [7]. Indigenous Peoples of the Northern Great Plains did not consume dairy foods as part of their traditional dietary intake [8]. Consistent with knowledge on dairy availability and consumption, many Indigenous Peoples display a reduced ability to digest lactose [9,10,11,12,13]. Lactase persistence, or the ability to continue to digest lactose beyond infancy, is a trait more prevalent among people of European ancestry than among other populations [14].

Dairy foods serve as an important source of calcium (Ca) in the contemporary American diet [15,16]. Approximately 1000 mg per day of Ca intake is recommended for most American adults [17]. Little is known about pre-contact Ca needs or intakes of Great Plains Indians. Some historic evidence for adequate nutriture exists as evidenced by tall stature among various Northern Great Plains tribes. Height is described as a metric of the “general health status of a population” but does not necessarily indicate whether other influences such as trauma or disease affected health [18]. Some sources indicate that Great Plains Native American men were the tallest in the world during the mid-1800s [18,19,20], which could indicate adequate nutrition, including sufficient Ca intake.

Current data indicates that Native Americans tend to consume less Ca than other ethnic groups in the United States [21,22,23]. Low Ca intake is considered by Western medical systems to be a known risk factor for osteoporosis [24]. Osteoporosis rates among Native Americans have not been extensively—or recently—investigated [24]. A 2005 study found that 11.9% of Native American women had osteoporosis, higher than any other ethnic group in the sample, and 44.5% had osteopenia [25]. Yet, after adjustment for covariates, Native American women were found to have a risk of osteoporosis similar to that experienced by white and Hispanic women [25,26]. Data from the National Health and Nutrition Examination Survey (NHANES) do not contain a large enough sample size of Native American participants to estimate current osteoporosis rates in this population [27,28].

Although dairy foods are a recommended part of DGA dietary patterns, it was not appropriate to include them in the TID developed for our intervention study (ClinicalTrials.gov Identifier: NCT06674642). Without dairy foods, the TID contained slightly more than half of the Recommended Dietary Allowance (RDA) for Ca [17,29]. The TID provides 586 mg out of 1000 mg/day RDA for females 19–50 y and males 19–70 y [29]. The Ca RDA was established using NHANES data as well, so it is not known if these recommendations are suited for the Native American population. Many NHANES analyses group Native Americans into an “Other race” racial category alongside Alaska Natives, Native Hawaiians, other Pacific Islanders, and multiracial individuals [30,31,32]. Ca has been classified as a nutrient of public health concern for all generally healthy Americans since 2005 [5,33,34,35].

### 1.1. Cultural Importance and Traditional Production of Culinary Ash

Since cattle were not utilized for dairy in North America until the late 1800s, we explored using juniper ash as a source of Ca in the intervention study diet as an alternative to dairy foods [36,37,38]. Juniper ash is a traditional food that has long been an important source of nutrients for indigenous North Americans. Most notably, in tribes located in southwest North America, juniper ash is added to a blue corn meal and water mixture to produce blue corn mush, bread, tamales, dumplings, and pancakes [39]. Juniper ash can also be mixed with hot water, strained, and incorporated into dough [39]. Other Indigenous tribes, such as the Hopi, have made culinary ash from the four-wing salt bush, spent bean vines and pods, corn cobs, and sheep dung, in addition to juniper branches [38]. This ash was used to make *piki*, a traditional Hopi bread and *bivlviki* or blue marbles/dumplings [40]. In addition to providing nutrition, juniper ash was also used as a flavoring agent and was used in traditional ceremonies, healing rituals, and as heat therapy for healing injured muscles [41].

According to oral accounts from a Navajo Traditional Consultant, historically, juniper ash was produced using a wood fire [42]. An entire juniper branch would be ignited then placed on a sheet of foil to burn undisturbed [42]. Once cooled, the ash was then collected and sifted. Branches with fewer berries were preferred, and lighter gray ash was favored over darker ash. If the ash is too dark in color, the process was repeated to ensure that a light-colored ash was achieved [42].

### 1.2. Juniper Ash Availability

As of this writing, juniper ash intended for human consumption can be purchased via e-commerce from independent vendors or online stores that carry Indigenous products. Previous literature analyzed the Ca content of juniper ash, reporting concentrations ranging from 125 mg/g to 309 mg/g [38,41,42,43]. Previous analyses of juniper ash have shown arsenic, lead, cadmium, and selenium below limits of detection (<1 ppm) in samples taken from 5 sites across the northern part of the Navajo Nation [41]. However, most of these analyses were conducted more than 30 years ago, and few reports have been published more recently on the nutrient content and safety of juniper ash for use in food. This project analyzed both freshly prepared and commercially available juniper ash samples, assessing their nutritive elements as well as their potentially toxic elements, to evaluate their potential as an alternative source of dietary Ca within the TID. Although no pesticide residues were expected to be present in the samples due to combustion, all samples were also analyzed for pesticide residues to ensure potential hazards to human health were identified.

## 2. Materials and Methods

To explore the potential use of juniper ash in the TID, we analyzed both commercially available juniper ash and juniper ash samples generated in our lab using juniper branches native to North Dakota (Supplemental Table S1). These samples were tested for a wide range of nutritive and potentially toxic trace elements (list below) as well as pesticides. Juniper ash available at retail was purchased online in February 2024 and stored in original packaging at room temperature until analysis. A detailed description of the methods used to generate juniper onsite is included below.

Juniper ash was analyzed for the following elements: aluminum (Al), antimony (Sb) arsenic (As), barium (Ba), beryllium (Be), boron (B), cadmium (Cd), Ca, chromium (Cr), cobalt (Co), copper (Cu), iron (Fe), lead (Pb), lithium (Li), magnesium (Mg), manganese (Mn), mercury (Hg), molybdenum (Mo), nickel (Ni), phosphorus (P), potassium (K), selenium (Se), silver (Ag), sodium (Na), strontium (Sr), thallium (Tl), tin (Sn), uranium (U), vanadium (V), and zinc (Zn).

### 2.1. Materials-North Dakota Juniper Samples

**Figure 1:**
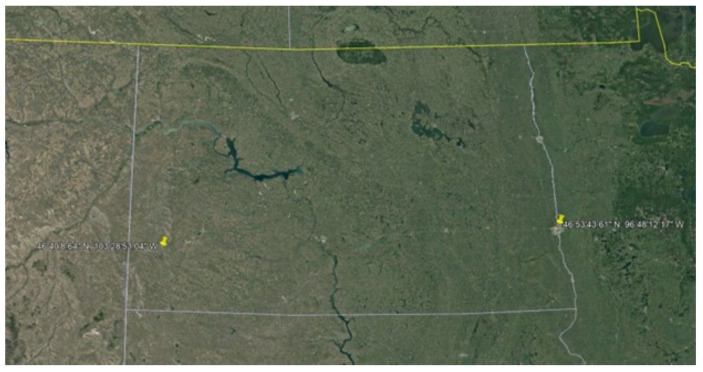
Geographic origins of North Dakota juniper branches used in analysis.

Two varieties of Juniper branches- Rocky Mountain Juniper (RMJ; *Juniperus scopulorum*) and Eastern Red Cedar (ERC; *Juniperus virginiana)-* were harvested from trees that were not sprayed or treated and located in two locations in North Dakota (ND). Figure 1 shows the geographic origins of the ND juniper samples. Both species are native to ND [44,45]. Branches were collected in spring 2023 and exposed to air at room temperature until bark was removed to allow the branches to dry fully. Branches—without foliage or berries—were selected for analysis based upon previous records indicating that the berries give a bitter taste to the ash [42].

ERC branches were largest in size and had a greater heartwood to sapwood ratio when compared to RMJ branches (Supplemental Tables S2 and S3). Heartwood, which serves as the tree’s supporting pillar, is dark in color, whereas sapwood, the tree’s pipeline for water to the leaves, is lighter in color and represents new wood in concentric growth [46]. Photographs of branches can be seen in Supplemental Figures S1–S4.

### 2.2. Methods: Ash Preparation

#### 2.2.1. Branch Preparation

After branches had been stored for 7 days, bark was removed. Loose bark was manually peeled back first. Then branches were shaved with utility knives to remove the remainder of the bark, working under a fume hood to mitigate dust. Samples were stored uncovered at room temperature to prevent moisture buildup. Separate weights of bark and branches were recorded (Supplemental Table S3). A keyhole saw was used to remove ridges and to cut branches too large to fit in a woodchipper. A commercial woodchipper (GUO054, GreatCircle Machinery, Ontario, CA, USA) was then used to break branches into smaller chips prior to drying and ashing the samples to ensure even distribution in crucibles. A clean garbage bag was fitted around a clean recycling bin to catch the chips from the spout of the woodchipper.

#### 2.2.2. Dehydration

Woodchips were weighed and spread evenly on a baking sheet. Small quantities were then placed into 100 mL food-safe porcelain crucibles (1883-05, ChemGlass, Vineland, NJ, USA) on baking sheets as representative samples to track dehydration rates (Supplementary Files). The branches were dehydrated in a commercial kitchen oven at ~90 °C in 8 h increments (Supplemental Figure S5). After each 8 h interval, representative samples were weighed to assess moisture loss. Dehydration processes continued until the volume remained unchanged after an 8 h dehydration cycle (Supplemental Table S4; Supplemental Figure S6).

*Ashing:* A 24 h ramping method (200 °C → 400 °C → 600 °C) was followed similar to previously completed analyses [42]. Crucibles containing juniper chips were placed in a furnace, which was ramped to 600 °C, over 4 h. The temperature was held for 24 h then the oven was allowed to cool. Two muffle furnaces (Thermolyne, Waltham, MA, USA) were utilized to ash juniper. Dehydrated woodchips in 25 g batches were placed into food-safe 500 mL porcelain crucibles (1883-05, -07, ChemGlass) prior to ashing (Supplemental Figure S7). Weights were recorded before and after the ashing process (Supplemental Table S5).

### 2.3. Methods: Ash Analysis

After the ashing process was complete, ash samples were combined to generate a single homogenous sample for each type of juniper assessed (RMJ and ERC).

Because juniper ash is used in culinary preparations, the weight of 1 teaspoon of ash was determined for each of the juniper ash types (RMJ, ERC, vendor-purchased). A teaspoon measure was first weighed (empty), then filled with ash and leveled at the top with a thin metal leveler [47]. The teaspoon containing the ash was then weighed, and the weight of the teaspoon measure subtracted to leave the weight of the ash alone. This process was repeated three times for each type of ash, and the average weights recorded [47].

Samples of RMJ, ERC, and vendor-supplied ash were sent in triplicate (analytical replicates from the same homogenized composite) to external laboratories for trace mineral analysis and pesticide residue analysis (Brooks Applied Labs (Seattle, WA, USA) and AGQ Labs (Oxnard, CA, USA), respectively). Samples of juniper ash purchased from an online vendor were also submitted for analysis of pesticide residues and trace mineral content.

*Pesticide Residues:* Ash samples were analyzed for 576 active ingredients by AGQ Laboratories following AOAC 2007.01 using gas chromatography-mass spectrometry (GC-MS) and liquid chromatography-mass spectrometry (LC-MS). A full list of pesticide residues assessed, and the type of instrument utilized for each one, can be found in Supplemental Files. Cucumber pesticide reference material was submitted with the juniper samples to AGQ Labs for the same analyses to serve as a standard (ERMBC403-0086, European Reference Standards [48]). Limit of quantification for each pesticide can be found in Supplemental Table S6.

*Nutritive and Toxic Elements:* A list of nutritive and toxic elements to be assessed in juniper ash was taken from the Food and Drug Administration’s Total Diet Study [49]. Nutritive elements included: Ca, Cu, Fe, K, Mg, Mn, Na, P, Se, and Zn. Toxic elements included: Ag, Al, As, Ba, Be, Cd, Co, Cr, Hg, Li, Mo, Ni, Pb, Sb, Sn, Sr, Tl, U, and V. B content was also analyzed. IEH Trace Metal and Metal Speciation Lab by Brooks Applied Labs analyzed the juniper ash samples.

Samples were digested prior to analysis with a modified USEPA 3051 digestion protocol [50] by weighing a known mass of each sample into Teflon digestion vessels with 4 mL HNO_3_, 1 mL H_2_O_2_, and 1 mL HCl. Vessels were sealed and heated in a laboratory microwave digestion system to 190 °C for at least 10 min. Resulting digests were cooled and diluted to a final known volume prior to analysis.

The sample digests were analyzed by triple quadrupole ICP-MS analysis (ICP-QQQ-MS) via a modified USEPA Method 6020 [51]. Each nutritive and nonnutritive element of interest was analyzed using multiple isotopes, when available. Multiple collection/reaction gases, including helium, hydrogen, oxygen, and ammonia, were used to ensure effective removal of polyatomic and isobaric interferences. Multiple internal standards including Ge, Rh, In, and Te were monitored during analyses and used to correct for instrument drift and/or matrix effects. A blank spike was prepared with the samples for quality control and met acceptance criteria for nutritive and nonnutritive elements analyzed (Supplemental Table S7). Method reporting limits for each analyte can be found in Supplemental Table S8.

## 3. Results

Final lab-generated ash products were light gray in appearance with flecks of orange, blue, and dark gray (Supplemental Figure S8). The texture was light and airy, sticking to the container via static electricity. Ash products from both juniper species displayed similar physical characteristics. Purchased ash was slightly darker in appearance than the lab-generated ash (Supplemental Figure S9).

### 3.1. Pesticide Analyses

None of the juniper ash samples tested positive for pesticide residues (Supplemental Files).

### 3.2. Nutritive and Toxic Elements

Amounts of nutritive and potentially toxic elements found in each sample are listed in Table 1, Table 2, Table 3, Table 4, Table 5 and Table 6. Amounts are recorded in mg/g or μg/g as well as amount per teaspoon to provide a clinically relevant measurement, as juniper ash is typically added to recipes in amounts ≤ 1 tsp per individual serving [41]. The average Ca provided per teaspoon of ash was 445 ± 141 mg. Per gram of ash, the Ca content averaged 331 ± 39.8 mg. There were also differences in average Fe (3.1 ± 2.7 mg per g), Cu (122 ± 45 μg per g), K (68 ± 25 mg), and Mg (17.6 ± 5.5 mg) contents of the different ash varieties. All analyses met quality control criteria and did not provide indications of contamination or bias.

All ash samples also contained potentially toxic elements and some toxic elements. For those elements that have a tolerable upper limit (UL; B, Mo, Ni, V) for human intake [52], the samples contained less than 5% of any UL per teaspoon of ash. Each analyzed samples contained Pb in amounts ranging from 2.6 ppm in RMJ to 1.09 ppm in ERC to 15 ppm in the vendor provided sample. There were also substantial differences in Al content, ranging from 0.19 mg/g in ERC to 1.7 mg/g in RMJ to 9.4 mg/g in the vendor sample, in the Li, Sr, and V content of the three juniper varieties as well (Table 4, Table 5 and Table 6). Some detectable amounts of each potentially toxic element analyzed except mercury were found in each of the juniper ash types. Hg (0.005 mg/kg) was only detected in the vendor-purchased ash and not in RMJ or ERC. Table 7 contains a comparison of the elemental composition of RMJ, ERC, and vendor-purchased ash and method detection limits for each analyte.

**Table 1:** Nutritive Analyses of Rocky Mountain Juniper Ash Sample.

Element	Quantity (Unit/g)	Quantity (Unit/tsp)	Dietary Recommendations for Males 19–30 y [16,53,54,55]	Percent of Dietary Recommendation Provided by 1 g Juniper Ash (%)	Tolerable Upper Limit (UL) Males and Females 19–50 y [52]
Ca (mg)	316	333.5	1000	32	2500
Cu (μg)	165	174.1	900	18	10,000
Fe (mg)	2.55	2.56	8	32	45
K (mg)	96.2	96.7	3400	3	ND *
Mg (mg)	23.9	25.2	400	6	350
Mn (mg)	0.503	0.53	2.3	22	11
Na (mg)	4.05	4.27	≤2300	0	ND
P (mg)	10.6	11.2	700	2	4000
Se (μg)	0.324	0.342	55	1	400
Zn (mg)	0.424	0.426	11	4	2500

* ND = Not determinable.

**Table 2:** Nutritive Analyses of Eastern Red Cedar Ash Sample.

Element	Quantity (Unit/g)	Quantity (Unit/tsp)	Dietary Recommendations for Males 19–30 y [16,53,54,55]	Percent of Dietary Recommendation Provided by 1 g Juniper Ash (%)	Tolerable Upper Limit (UL) Males and Females 19–50 y [52]
Ca (mg)	377	397.9	1000	38	2500
Cu (μg)	127	134.04	900	14	10,000
Fe (mg)	0.664	0.668	8	8	45
K (mg)	49.7	52.4	3400	1	ND *
Mg (mg)	13.8	14.6	400	3	350
Mn (mg)	0.259	0.273	2.3	11	11
Na (mg)	1.35	1.42	≤2300	0	ND *
P (mg)	11.3	11.9	700	2	4000
Se (μg)	0.394	0.416	55	1	400
Zn (mg)	0.465	0.468	11	4	2500

* ND = Not determinable.

**Table 3:** Nutritive Analyses of Vendor-Purchased Ash Sample.

Element	Quantity (Unit/g)	Quantity (Unit/tsp)	Dietary Recommendations for Males 19–30 y [16,53,54,55]	Percent of Dietary Recommendation Provided by Juniper Ash (%)	Tolerable Upper Limit (UL) Males and Females 19–50 y [52]
Ca (mg)	302	603.5	1000	30	2500
Cu (μg)	74.6	149.08	900	8	10,000
Fe (mg)	5.98	12.0	8	75	45
K (mg)	58.1	116.1	3400	2	ND *
Mg (mg)	15.2	30.4	400	4	350
Mn (mg)	0.377	0.753	2.3	16	11
Na (mg)	1.23	2.46	≤2300	0	ND *
P (mg)	11.3	22.6	700	2	4000
Se (μg)	0.397	0.793	55	1	400
Zn (mg)	0.232	0.464	11	2	2500

* ND = Not determinable.

**Table 4:** Potentially toxic Analyses of Rocky Mountain Juniper Ash Sample.

Element	Quantity (mg/kg)	Quantity (Unit/tsp)	Tolerable Upper Limit (UL)	% ULs (per tsp)
Ag	0.317			
Al	1710			
As	0.504			
B	572	0.604	20	3.02%
Ba	3630			
Be	0.098			
Cd	0.075			
Co	4.77			
Cr	3.37			
Hg	0.0			
Li	38.6			
Mo	1.9	2.005	2000	0.10%
Ni	3.45	0.004	1.0	0.36%
Pb	2.6			
Sb	0.113			
Sn	0.686			
Sr	1120			
Tl	0.015			
U	0.163			
V	3.72	0.004	1.8	0.22%

**Table 5:** Potentially toxic Analyses of Eastern Red Cedar Ash Sample.

Element	Quantity (mg/kg)	Quantity (Unit/tsp)	Tolerable Upper Limit (UL) Males and Females 19–50 y	% UL (per tsp)
Ag	0.103			
Al	192			
As	0.203			
B	329	0.347	20	1.74%
Ba	1640			
Be	0.016			
Cd	0.027			
Co	1.95			
Cr	1.17			
Hg	0.0			
Li	17.1			
Mo)	2.38	2.512	2000	0.13%
Ni	0.883	0.001	1.0	0.09%
Pb	1.09			
Sb	0.049			
Sn	0.629			
Sr	355			
Tl	0.006			
U	0.026			
V	0.589	0.001	1.8	0.03%

**Table 6:** Potentially toxic Analyses of Vendor-Purchased Ash Sample.

Element	Quantity (mg/kg)	Quantity (Unit/tsp)	Tolerable Upper Limit (UL) Males and Females 19–50 y	% UL (per tsp)
Ag	0.041			
Al	9430			
As	1.51			
B	175	0.350	20	1.75%
Ba	263			
Be	0.339			
Cd	0.072			
Co	2.77			
Cr	6.75			
Hg	0.005			
Li	6.31			
Mo	7.26	14.508	2000	0.73%
Ni	4.72	0.009	1.0	0.94%
Pb	15			
Sb	0.567			
Sn	0.822			
Sr	3630			
Tl	0.063			
U	0.434			
V	15.3	0.031	1.8	1.70%

**Table 7:** Comparison of elemental composition of Rocky Mountain Juniper (RMJ), Eastern Red Cedar (ERC), and Vendor-supplied juniper ash samples and Method Detection Limits (MDL).

Element	RMJ (mg/kg)	MDL (mg/kg)	ERC (mg/kg)	MDL (mg/kg)	Vendor-Supplied Ash (mg/kg)	MDL (mg/kg)	Average Elemental Composition (mg/kg ± SD)
Ag	0.317	0.0008	0.103	0.0008	0.041	0.0008	0.15 ± 0.14
Al	1710	0.802	192	0.834	9430	162	3777.33 ± 4953.84
As	0.504	0.002	0.203	0.002	1.51	0.002	0.74 ± 0.68
B	572	40.1	329	41.7	175	4.05	358.67 ± 200.16
Ba	3630	0.716	1640	0.745	263	0.724	1844.33 ± 1692.77
Be	0.098	0.006	0.016	0.006	0.339	0.006	0.15 ± 0.17
Ca	316	783	377	815	302	791	331.67 ± 39.88
Cd	0.075	0.002	0.027	0.002	0.072	0.002	0.06 ± 0.03
Co	4.77	0.005	1.95	0.005	2.77	0.005	3.16 ± 1.45
Cr	3.37	0.048	1.17	0.05	6.75	0.048	3.76 ± 2.81
Cu	0.17	5.73	0.127	5.96	0.075	5.79	0.12 ± 0.05
Fe	2.55	68.8	0.664	71.5	5.98	69.5	3.06 ± 2.70
Hg	0	0.003	0	0.003	0.005	0.003	0.00 ± 0.00
K	96.2	60.2	49.7	62.6	58.1	60.8	68.00 ± 24.78
Li	38.6	0.008	17.1	62.6	6.31	0.008	20.67 ± 16.44
Mg	23.9	115	13.8	119	15.2	116	17.63 ± 5.47
Mn	0.503	1.91	0.259	1.99	0.377	1.93	0.38 ± 0.12
Mo	0.002	0.005	0.0024	0.005	0.0073	0.965	0.00 ± 0.00
Na	4.05	1150	1.35	5.96	1.23	5.79	2.21 ± 1.59
Ni	3.45	0.049	0.883	0.051	4.72	0.049	3.02 ± 1.95
P	10.6	9.55	11.3	9.93	11.3	9.65	11.07 ± 0.40
Pb	2.6	0.002	1.09	0.002	15	0.463	6.23 ± 7.63
Sb	0.113	0.002	0.049	0.002	0.567	0.002	0.24 ± 0.28
Se	0.0003	0.005	0.0004	0.005	0.0004	0.005	0.00 ± 0.00
Sn	0.686	0.002	0.629	0.002	0.822	0.002	0.71 ± 0.10
Sr	1120	3.82	355	3.97	3630	3.86	1701.67 ± 1713.23
Tl	0.015	0.003	0.006	0.003	0.063	0.003	0.03 ± 0.03
U	0.163	0.002	0.026	0.002	0.434	0.002	0.21 ± 0.21
V	3.72	0.002	0.589	0.002	15.3	0.002	6.54 ± 7.75
Zn	0.424	42	0.465	43.7	0.232	42.4	0.37 ± 0.12

## 4. Discussion

Juniper ash does contain some nutritive minerals and no pesticide residues, but the content of toxic elements raises concern about potential adverse health impacts from consumption. Because organic material is destroyed in the burning process, the finding of no pesticide residues was expected. The amount of Pb found in the analyzed samples, especially the vendor sample, was surprising, as the 1998 publication on this topic found no detectable amounts of Pb in juniper ash from the Navajo Nation. [41] The vendor who supplied the juniper ash for this study is also based within the Navajo Nation; however, the geographical origin of the juniper used to produce the ash is not known.

Due to the lack of scientific literature on this traditional food, its uses, the nutrition status of people who consume it regularly, and its interactions within a food matrix, it is difficult to appropriately contextualize the findings on the potentially toxic and toxic elements found within the juniper ash samples. For instance, the amount of Pb in the vendor-provided sample (15 ppm) exceeds the Food and Drug Administration (FDA)’s action level of 0.1 ppm for chocolate and sugar-based candy intended for consumption by children [56]. In Indigenous cooking traditions, juniper ash is considered a culinary seasoning [57]. However, beyond an action limit of 10 mg/kg for lead in “natural-source food color additives” such as paprika, saffron, and turmeric [58], there are no other permissible limits or action levels for lead established for most seasonings and spices in the U.S., and spices are not generally labeled as a food consumed by children [58].

It is also not known how toxic the Pb content of juniper ash may be. Gastrointestinal absorption of Pb is decreased by “the presence of food in the gastrointestinal tract” [59]. Because juniper ash is mixed into food before consumption, it is not known how the Pb content in the ash interacts with the rest of food matrix. Juniper ash also contains Ca and some Fe, both of which mitigate Pb absorption [59]. However, without research focused on the in vitro gastrointestinal bioaccessibility of Ca and Pb in juniper ash when incorporated into food, it is not possible to know how juniper ash may interact with a food matrix to affect in vivo uptake of these elements.

All juniper ash samples (RMJ, ERC, and retail) meet the food standard of being “high in” Ca [17], providing between 300 and 600 mg of Ca per teaspoon of ash (Table 4, Table 5 and Table 6), equal to about 33 to 60% of the RDA. The high standard deviation in average Ca content (445 ± 141 mg) is due to the greater amount of Ca provided per teaspoon by the vendor provided ash sample compared to RMJ and ERC. The vendor ash (1.99 g/tsp) is denser than the lab-generated ash (1.06 g/tsp). Although this study utilized laboratory methods to ash the juniper branches rather than traditional methods (outside over a flame), previous work comparing the Ca content of juniper ashed in a laboratory setting versus with traditional methods indicated a higher Ca concentration among the laboratory-ashed samples [42]. Begay et al. noted that laboratory ashed samples had a mean Ca concentration of 289 ± 30.6 mg/g compared to traditionally ashed samples with a mean Ca concentration of 242 ± 28.2 mg/g, and these differences were statistically significant [42].

The bioavailability of the Ca found in juniper ash is also unknown. Previous analyses indicate that the form of Ca found in juniper ash is Ca oxide (CaO) [41,42]. The European Food Safety Authority [60] permits CaO to be used in foods and supplements, and the FDA [61] recognizes CaO as generally safe when used in accordance with good manufacturing or feeding practices. It is unknown how bioavailable the Ca content of CaO might be.

## 5. Limitations

The juniper ash utilized for analysis represents a convenience sample, and reasons for the mineral content differences among the ash samples were not explored. Differences in nutritive and potentially toxic elements found in the ND-sourced juniper and the purchased juniper ash could be due to geographical origin of the juniper samples or processing methods. Insufficient information is available to estimate juniper ash consumption rates and, therefore, to estimate potential harm. While NHANES data is used to estimate daily spice and seasoning intake by Americans to determine potential risk of harm [62], it does not collect enough data on Native Americans to enable researchers to estimate health risks from spice intake specific to this population.

The yield of ash from the ND samples was too small to allow for additional samples from the same lot to be sent to other laboratories for a comparison analysis or to also have the iodine content of the samples analyzed. The RMJ generated 7 g of ash per kg of raw material, and the ERC generated 10 g of ash per kg of raw material. Initially, juniper ash was explored for potential inclusion as a source of Ca in the TID; however, generating the amount of ash needed for an intervention study would have taken years of work to produce.

One limitation to the finding of pesticide residues is that the results for the cucumber pesticide standard received from the testing lab exceeded the certified values [63]. All pesticides in the reference material were successfully detected, however.

Finally, according to Lillie Pete, the Navajo Traditional Consultant interviewed by Begay et al. [42], the best time to gather juniper for ashing is during the fall when red ends appear on juniper branches to ensure maximum flavor and nutrition. The juniper collected for our study was collected in July 2023. Due to timing of the planned feeding study, we were unable to delay juniper branch collection until the fall months. However, the timing of juniper collection may limit the applicability of these findings to traditionally produced juniper ash.

## 6. Conclusions

Information on juniper ash, even with these analyses, is too sparse to determine whether it meets contemporary standards for food safety and can be included in an intervention diet as part of a healthy eating pattern. Too little is understood about the bioavailability of CaO, including how that bioavailability may be affected by the typical food matrix in which juniper ash is served (that is, blue corn-based dishes and meals), to determine whether juniper ash adds or detracts from nutrition status.

To our knowledge, this study is the first evaluation of the elemental composition of juniper ash published in the peer-reviewed literature since 1998 [41]. The 1998 study [41] reported that As, Pb, Cd, and Se levels in juniper was harvested from the Four Corners area (junction of Utah, Colorado, Arizona, and New Mexico) of the Red Mesa Navajo Nation were below 1 ppm (the limits of detection). Bioavailability of Ca and other elements in juniper ash and in food preparations including juniper ash have not been evaluated. Burgeoning interest in plant-based sources of dietary Ca as well as a recent focus on Indigenous nutrition in the field of public health indicate that this topic may warrant further investigation.

Future research would need to sample juniper ash from different geographic regions, especially the Navajo Nation, where use of juniper ash is most often reported, to understand variability in mineral composition and potential safety ramifications of juniper ash use. In addition, the bioavailability of the elements in juniper ash, especially Ca and Pb, need to be evaluated with in vitro digestion models to assess both safety and potential benefits of juniper ash consumption. Finally, given the dearth of information on current micronutrient intakes of the Indigenous population in the U.S., these populations should be oversampled in future nationally representative dietary intake studies so that nutrient shortfalls and needs of this population can be better understood and addressed.

## Data Availability

The original contributions presented in this study are included in the article/Supplementary Material. Further inquiries can be directed to the corresponding author.

## Supplementary Materials

The following supporting information can be downloaded at: https://www.mdpi.com/article/10.3390/nu18020260/s1, Figure S1. Rocky Mountain Juniper branches (large) after bark removal. Figure S2. Eastern Redcedar Juniper branches (medium) after bark removal. Supplemental. Figure S3. Eastern Redcedar Juniper branches (small) after bark removal. Figure S4. Small Eastern Redcedar and Rocky Mountain Juniper specimens after bark removal. Figure S5. Dehydration process of juniper ash woodchips with crucible for scale. Figure S6. Dehydrated juniper woodchips. Figure S7. Crucibles filled with juniper woodchips in furnace. Figure S8. Laboratory-made juniper ash close-up. Figure S9. Appearance of vendor-purchased juniper ash. Table S1. Source of Juniper Ash used in Analysis. Table S2. Diameters of Branches (after bark removal). Table S3. Initial Bark and Wood Weights. Table S4. Weights before and after dehydration process. Table S5. Juniper weights before and after burning process. Table S6. Pesticides included in Juniper Ash Analysis. Table S7. Accuracy and Precision Summary from Blank Spike and Recovery Data. Table S8. Method Reporting Limits for Juniper Ash Samples (n = 3).

## Abbreviations

Agricultural Research Service (ARS), aluminum (Al), antimony (Sb), arsenic (As), barium (Ba), beryllium (Be), boron (B), cadmium (Cd), calcium (Ca), chromium (Cr), cobalt (Co), copper (Cu), Dietary Guidelines for Americans (DGA), Eastern Redcedar (ERC), Food and Drug Administration (FDA), Grand Forks Human Nutrition Research Center (GFHNRC), iron (Fe), lead (Pb), lithium (Li), magnesium (Mg), manganese (Mn), mercury (Hg), molybdenum (Mo), nickel (Ni), North Dakota (ND), phosphorus (P), potassium (K), Recommended Dietary Allowance (RDA), Rocky Mountain Juniper (RMJ), selenium (Se), silver (Ag), sodium (Na), strontium (Sr), thallium (Tl), tin (Sn), Traditional Indigenous Diet (TID), United States Department of Agriculture (USDA), uranium (U), vanadium (V), zinc (Zn).
