# Supplementary material for: Assessment of Juniper Ash Elemental Composition for Potential Use in a Traditional Indigenous Dietary Pattern
Source: Nutrients. 2026 Jan 14;18(2):260. doi: 10.3390/nu18020260 (PMC12844745; doi:10.3390/nu18020260)
Supplement: Supplementary file 1 [file nutrients-18-00260-s001.zip › nutrients-4076957-supplementary.pdf]

Hess et al.— Assessment of Juniper Ash Elemental Composition for Potential Use in a Traditional Indigenous Dietary Pattern

# Supplemental Figures and Tables

# Supplemental Figures

## Supplemental Figure 1. Rocky Mountain Juniper branches (large) after bark removal

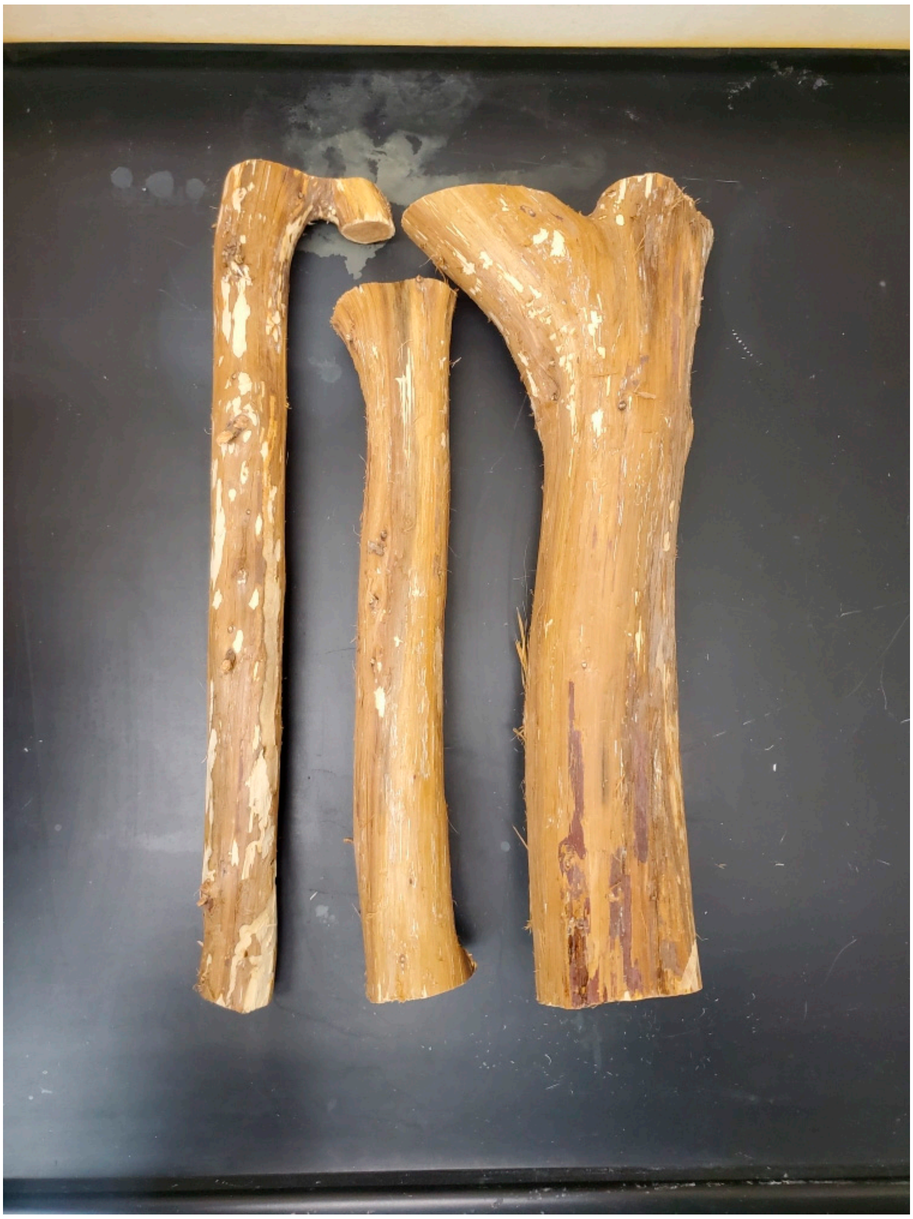

## Supplemental Figure 2. Eastern Redcedar Juniper branches (medium) after bark removal

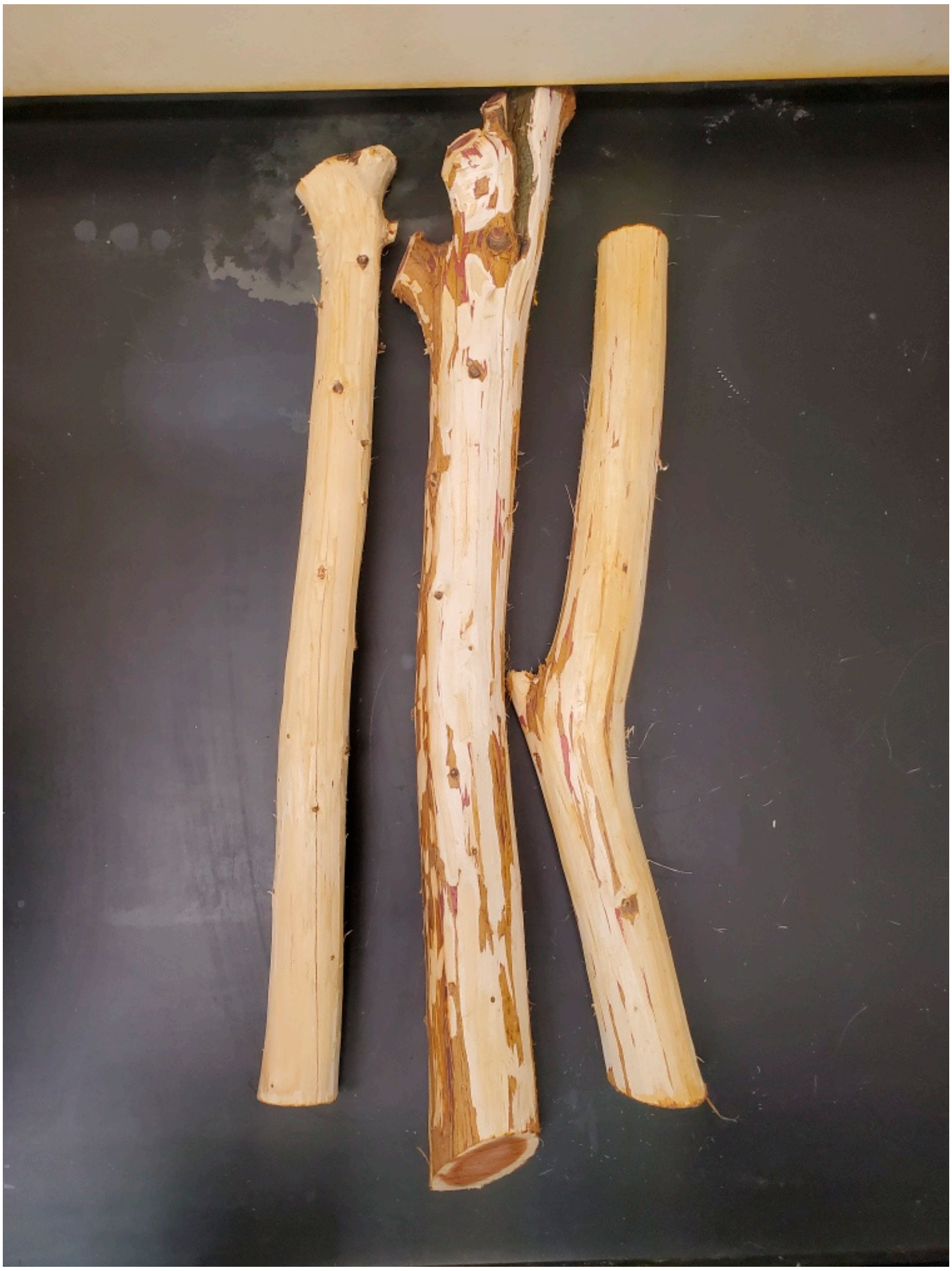

## Supplemental Figure 3. Eastern Redcedar Juniper branches (small) after bark removal

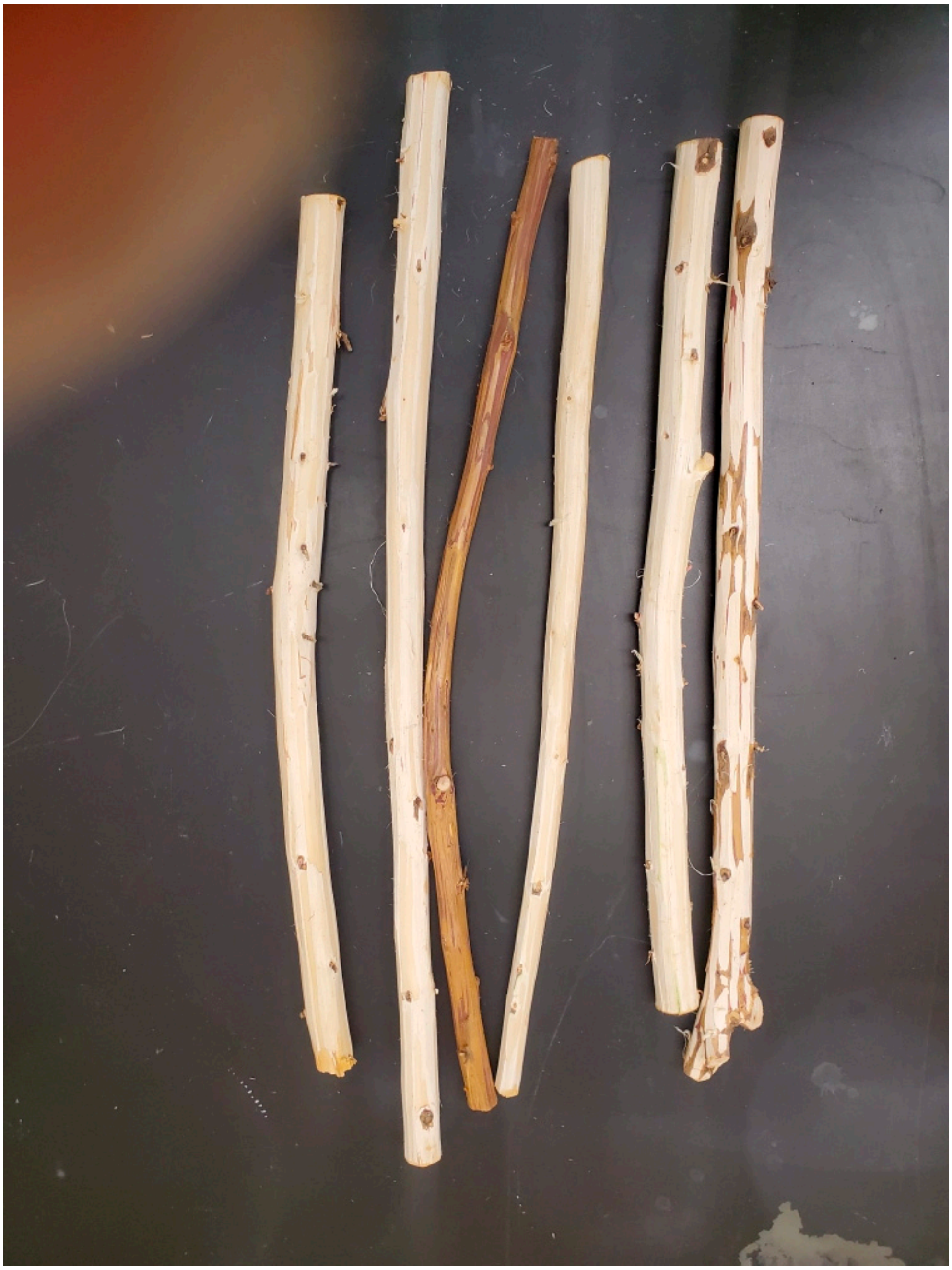

## Supplemental Figure 4. Small Eastern Redcedar (from left, first and second specimens) and Rocky Mountain Juniper (third from left) specimens after bark removal

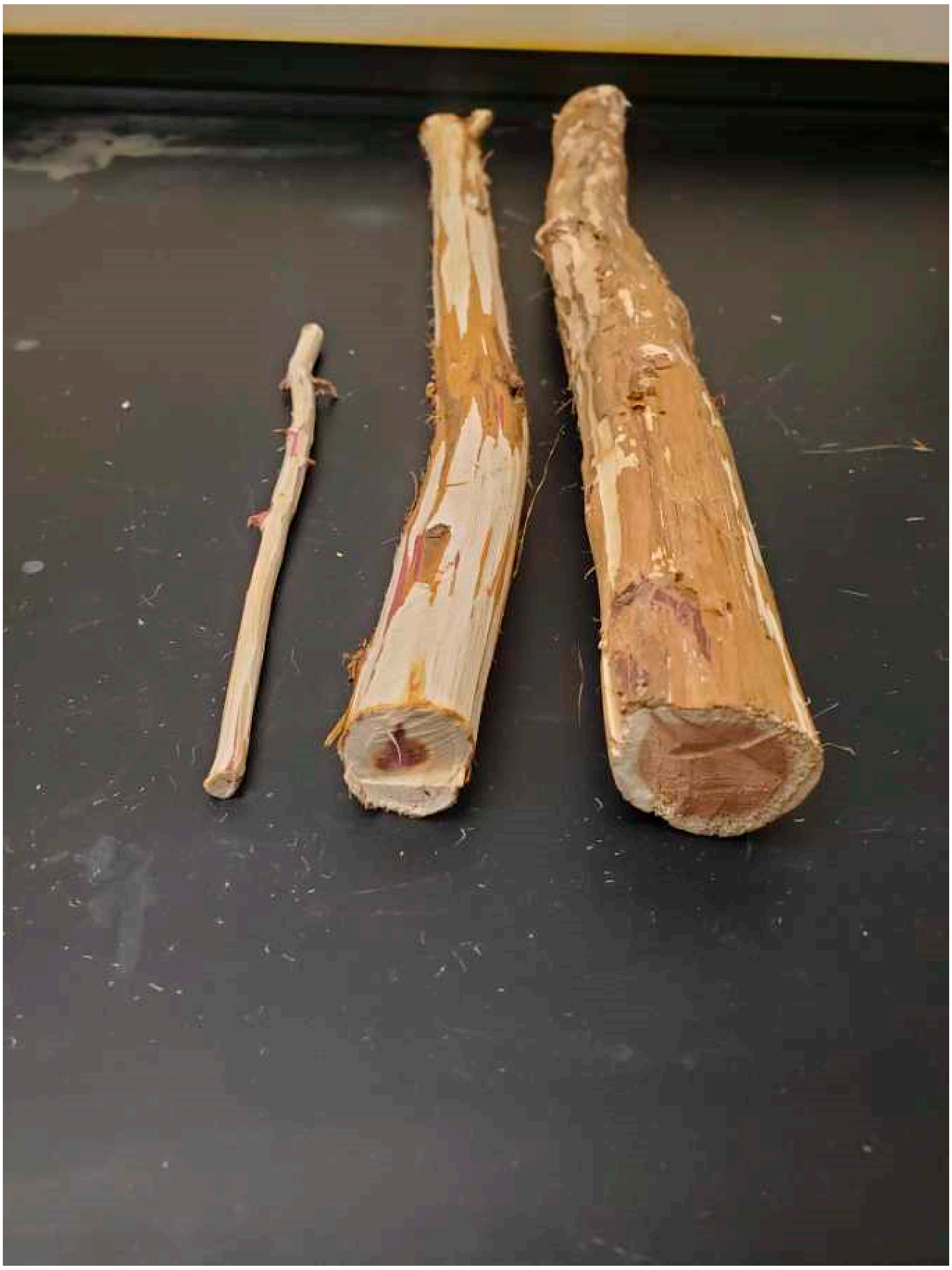

## Supplemental Figure 5. Dehydration process of juniper ash woodchips with crucible for scale

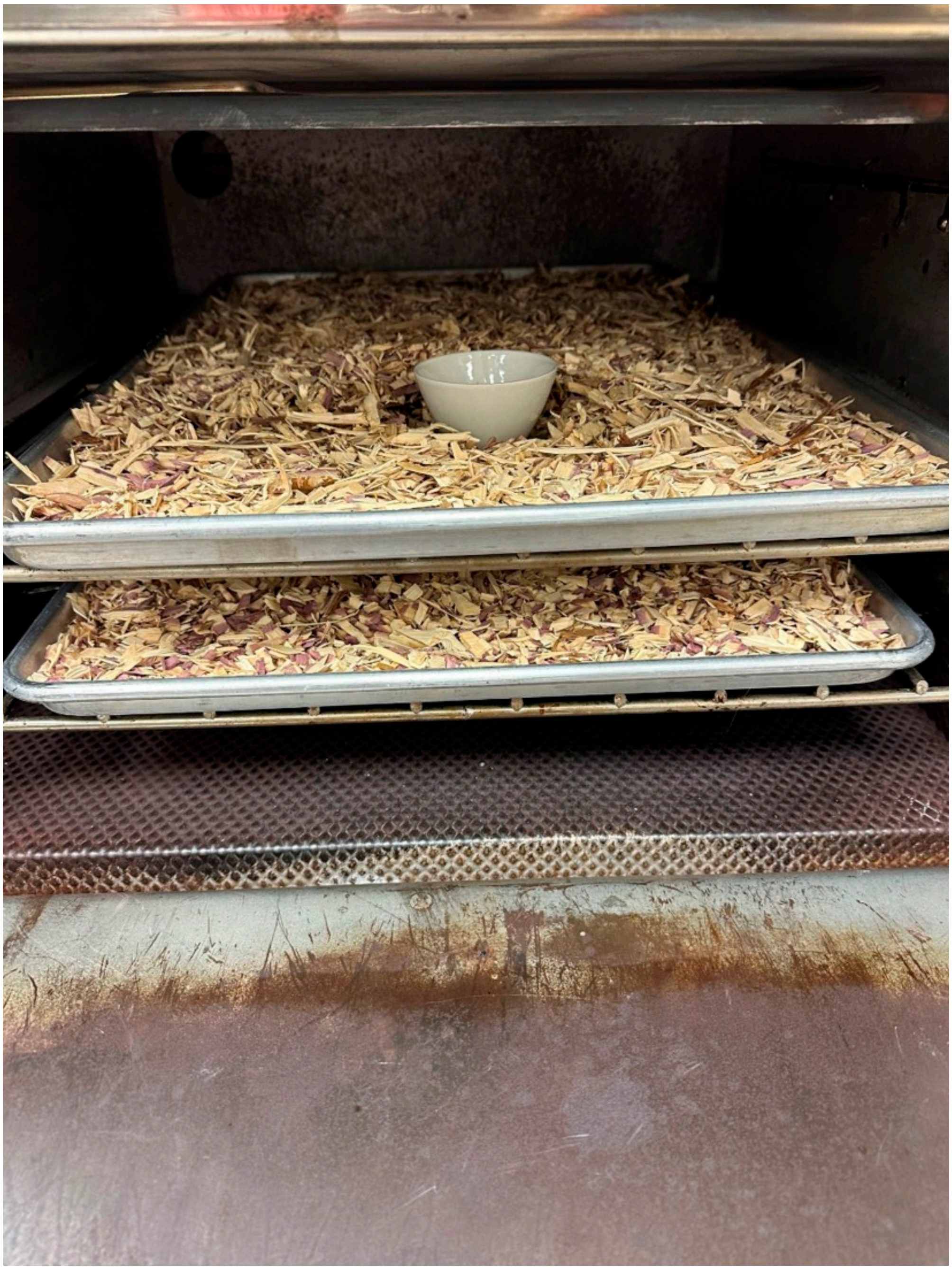

## Supplemental Figure 6. Dehydrated juniper woodchips

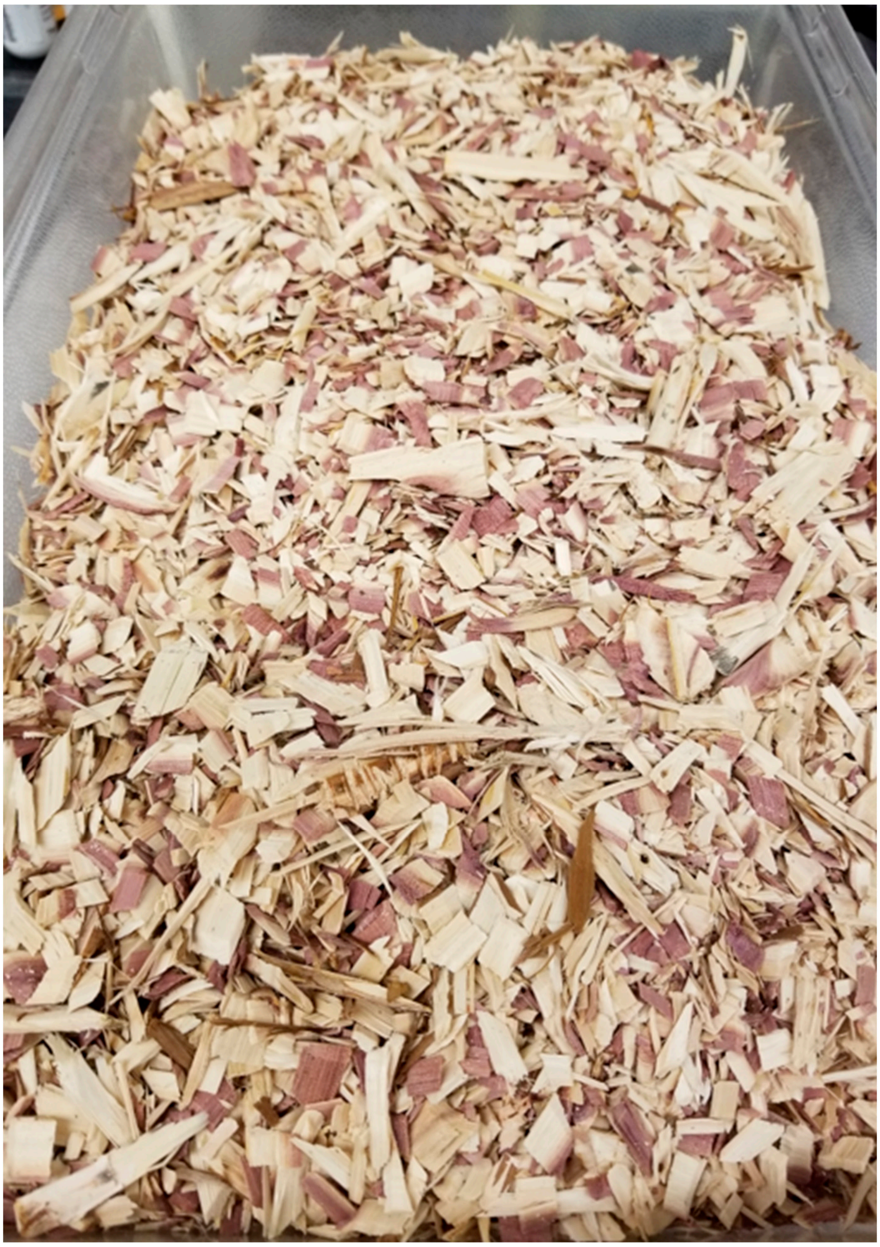

## Supplemental Figure 7. Crucibles filled with juniper woodchips in furnace

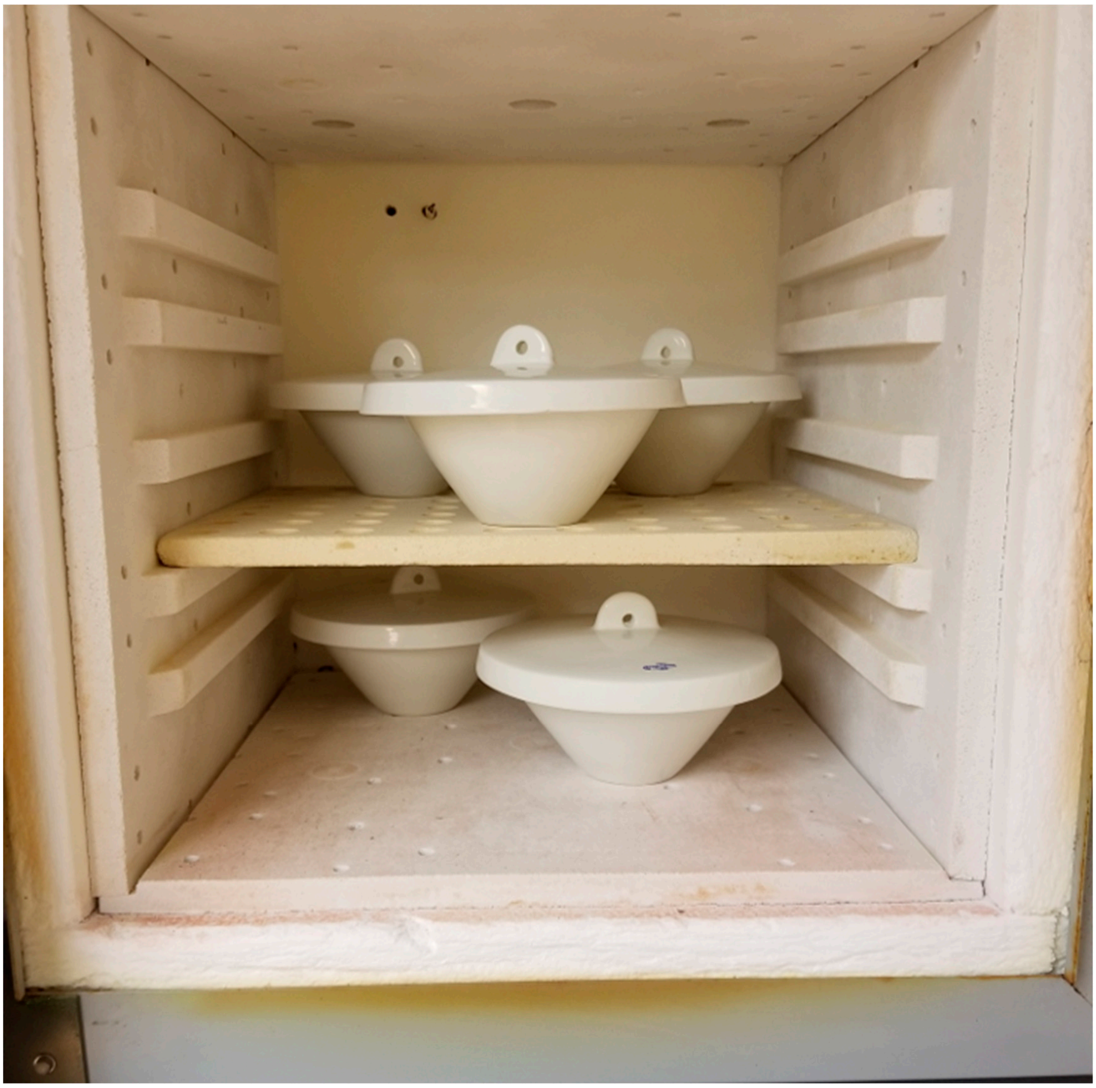

## Supplemental Figure 8. Laboratory-made juniper ash close-up

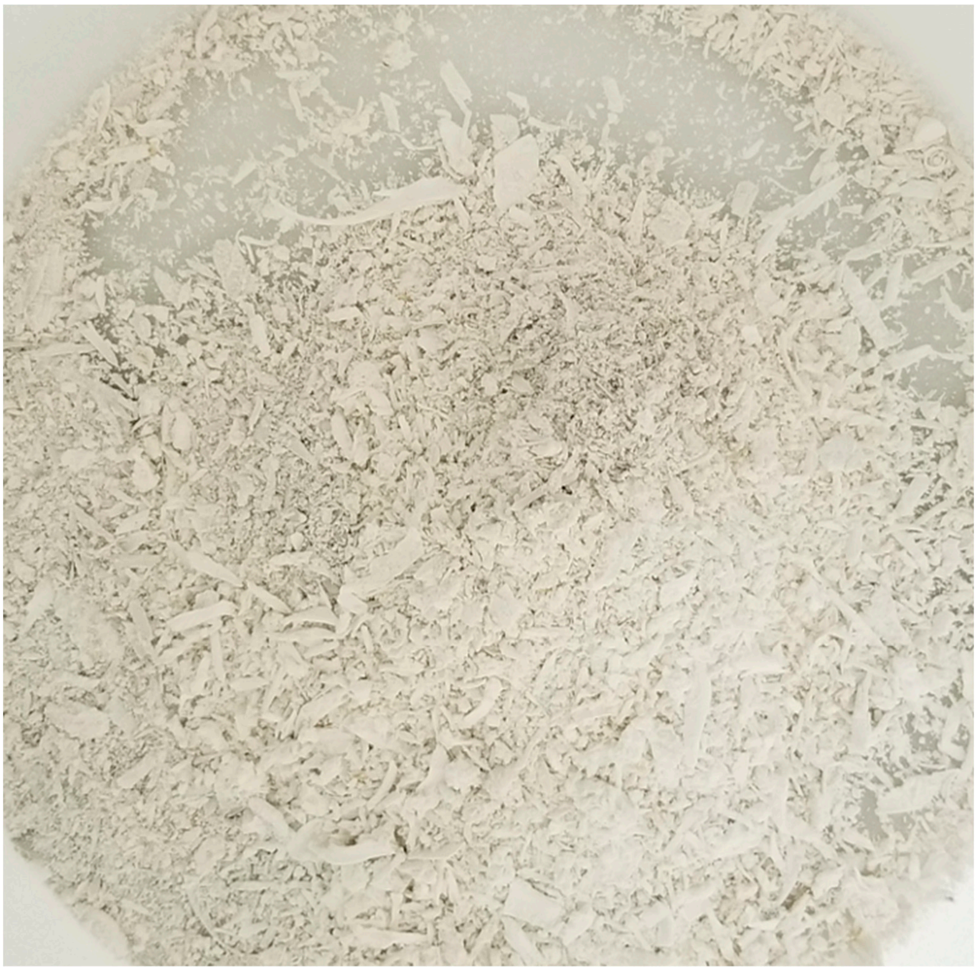

## Supplemental Figure 9. Appearance of vendor-purchased juniper ash

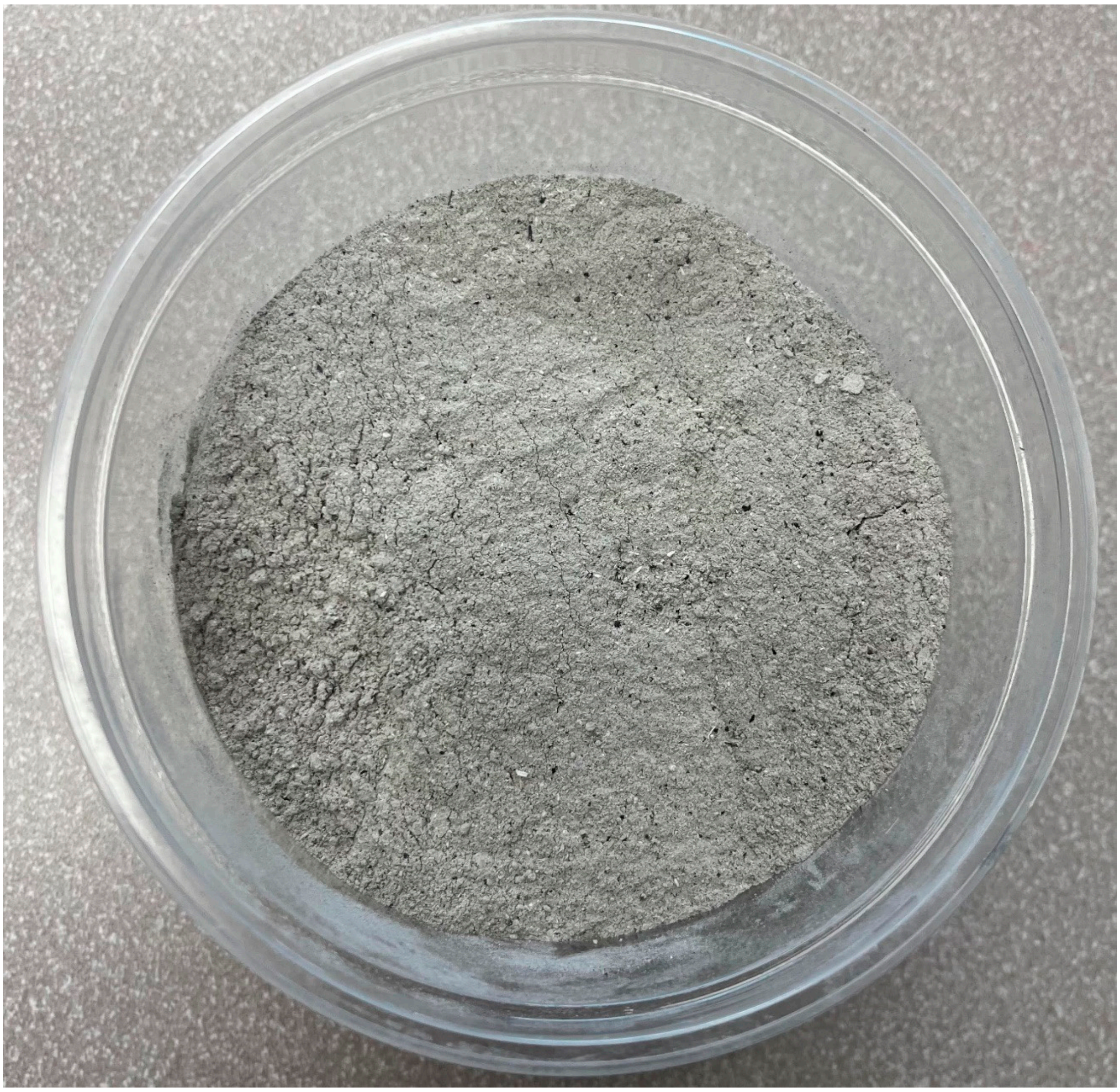

Hess et al.— Assessment of Juniper Ash Elemental Composition for Potential Use in a Traditional Indigenous Dietary Pattern

# Supplemental Tables

## Supplemental Table 1. Source of Juniper Ash used in Analysis

| ***Genus and Species*** | ***Juniperus virginiana*** | ***Juniperus scopulorum*** |
|---:|---:|---:|
| *Geographic location* | Southeastern North Dakota | Western North Dakota |
| *Status* | Most branches alive upon collection | Dead upon collection |

## Supplemental Table 2. Diameters of Branches (after bark removal)

| | **Rocky Mountain Juniper (largest branches)** | | **Eastern Redcedar (medium branches)** | | **Eastern Redcedar (smallest branches)** | |
|---|---|---|---|---|---|---|
| | Total | Heartwood | Total | Heartwood | Total | Heartwood |
| *Largest (cm)* | 7 | 5.6 | 5.7 | 4.6 | 1.4 | 0.3 |
| *Smallest (cm)* | 3 | 2.3 | 2.3 | 1 | 0.6 | 0 |

| | **Rocky Mountain Juniper (largest branches)** | | **Eastern Redcedar (medium branches)** | | **Eastern Redcedar (smallest branches)** | |
|---|---|---|---|---|---|---|
| | Largest | Smallest | Largest | Smallest | Largest | Smallest |
| *Heartwood (cm)* | 5.6 | 2.3 | 4.6 | 1 | 0.3 | 0 |
| *Total (cm)* | 7 | 3 | 5.7 | 2.3 | 1.3 | 0.6 |
| ***Percentage of Heartwood to Total (%)*** | **80** | **77** | **81** | **43** | **21** | **0** |

## Supplemental Table 3. Initial Bark and Wood Weights

| | **Rocky Mountain Juniper (largest branches)** | **Eastern Redcedar (medium branches)** | **Eastern Redcedar (smallest branches)** |
|---|---|---|---|
| *Raw weight (g)* | ≈2497.16 | ≈3275.79 | 902.17 |
| *Bark weight (g)* | 551.68 | 398.79 | 192.88 |
| *End weight (g)* | 1945.48 | 2877 | 710.53 |

## Supplemental Table 4. Weights before and after dehydration process

| | **Rocky Mountain Juniper (largest branches)** | **Eastern Red Cedar (small and medium branches)** |
|---|---|---|
| *Raw weight (g)* | 1945.48 | 3587.53 |
| *End weight (g)* | 1093.53 | 3181.54 |
| *Total water loss (g)* | 851.95 | 405.99 |

## Supplemental Table 5. Juniper weights before and after burning process

| | **Rocky Mountain Juniper (largest branches)** | **Eastern Red Cedar (small and medium branches)** |
|---|---|---|
| *Raw weight (g)* | 1093.53 | 3181.54 |
| *End weight (g)* | 12.49 | 30.11 |

## Supplemental Table 6. Pesticides included in Juniper Ash Analysis

| Parameter | Technique/ Instrument | Rocky Mountain Juniper Ash (mg/kg) | Eastern Red Cedar Ash (mg/kg) | Eastern Red Cedar Ash 2 (mg/kg) | Retail Juniper Ash (mg/kg) | Limit of Quantitation |
|---|---|---|---|---|---|---|
| 2,4,6-Trichloroanisole | GC-MS/MS | <0.010 | <0.010 | <0.010 | <0.010 | 0.010 |
| 2,4,6-Trichlorophenol | GC | <0.010 | <0.010 | <0.010 | <0.010 | 0.010 |
| 2-phenylphenol (SP) | GC | <0.010 | <0.010 | <0.010 | <0.010 | 0.010 |
| 3-OH carbofuran (SQ) | LC | <0.010 | <0.010 | <0.010 | <0.010 | 0.010 |
| 8-hydroxyquinoline (SP) | GC | <0.010 | <0.010 | <0.010 | <0.010 | 0.010 |
| Abamectin | LC | <0.010 | <0.010 | <0.010 | <0.010 | 0.010 |
| Acephate | LC | <0.010 | <0.010 | <0.010 | <0.010 | 0.010 |
| Acequinocyl | LC | <0.010 | <0.010 | <0.010 | <0.010 | 0.010 |
| Acetamiprid | LC | <0.010 | <0.010 | <0.010 | <0.010 | 0.010 |
| Acetochlor | GC | <0.010 | <0.010 | <0.010 | <0.010 | 0.010 |
| Acibenzolar-S-methyl | LC | <0.010 | <0.010 | <0.010 | <0.010 | 0.010 |
| Acrinathrin | GC | <0.010 | <0.010 | <0.010 | <0.010 | 0.010 |
| Alachlor | GC | <0.010 | <0.010 | <0.010 | <0.010 | 0.010 |
| Aldicarb (SP) | LC | <0.010 | <0.010 | <0.010 | <0.010 | 0.010 |
| Aldicarb (sum) | LC | <0.010 | <0.010 | <0.010 | <0.010 | 0.010 |
| Aldicarb-sulfone | LC | <0.010 | <0.010 | <0.010 | <0.010 | 0.010 |
| Aldicarb-sulfoxide | LC | <0.010 | <0.010 | <0.010 | <0.010 | 0.010 |
| Aldrin | GC | <0.010 | <0.010 | <0.010 | <0.010 | 0.010 |
| Alpha Endosulfan | GC | <0.010 | <0.010 | <0.010 | <0.010 | 0.010 |
| Alph-HCH | GC | <0.010 | <0.010 | <0.010 | <0.010 | 0.010 |
| Ametoctradin | LC | <0.010 | <0.010 | <0.010 | <0.010 | 0.010 |
| Ametryn | GC | <0.010 | <0.010 | <0.010 | <0.010 | 0.010 |
| Aminocarb | LC | <0.010 | <0.010 | <0.010 | <0.010 | 0.010 |
| Amitraz (SP) | LC | <0.010 | <0.010 | <0.010 | <0.010 | 0.010 |
| Anthraquinone | GC | <0.010 | <0.010 | <0.010 | <0.010 | 0.010 |
| Atrazine | GC | <0.010 | <0.010 | <0.010 | <0.010 | 0.010 |
| Atrazine Desisopropyl | LC | <0.010 | <0.010 | <0.010 | <0.010 | 0.010 |
| Azaconazole | LC | <0.010 | <0.010 | <0.010 | <0.010 | 0.010 |
| Azadirachtin | LC | <0.010 | <0.010 | <0.010 | <0.010 | 0.010 |
| Azamethiphos | LC | <0.010 | <0.010 | <0.010 | <0.010 | 0.010 |
| Azimsulfuron | LC | <0.010 | <0.010 | <0.010 | <0.010 | 0.010 |

| | | | | | | |
|---|---|---|---|---|---|---|
| Azinphos-ethyl | LC | <0.010 | <0.010 | <0.010 | <0.010 | 0.010 |
| Azinphos-methyl | LC | <0.010 | <0.010 | <0.010 | <0.010 | 0.010 |
| Azocyclotin and Cyhexatin (SQ) | LC | <0.010 | <0.010 | <0.010 | <0.010 | 0.010 |
| Azoxystrobin | LC | <0.010 | <0.010 | <0.010 | <0.010 | 0.010 |
| Beflubutamid | GC | <0.010 | <0.010 | <0.010 | <0.010 | 0.010 |
| Benalaxyl | GC | <0.010 | <0.010 | <0.010 | <0.010 | 0.010 |
| Ben-Carb-TPM (Sum) | LC | <0.010 | <0.010 | <0.010 | <0.010 | 0.010 |
| Bendiocarb | LC | <0.010 | <0.010 | <0.010 | <0.010 | 0.010 |
| Benfluralin | GC | <0.010 | <0.010 | <0.010 | <0.010 | 0.010 |
| Bentazone (SP) | LC | <0.010 | <0.010 | <0.010 | <0.010 | 0.010 |
| Bentazones-methyl | LC | <0.010 | <0.010 | <0.010 | <0.010 | 0.010 |
| Benthiavalicarb | LC | <0.010 | <0.010 | <0.010 | <0.010 | 0.010 |
| Beta Endosulfan | GC | <0.010 | <0.010 | <0.010 | <0.010 | 0.010 |
| Beta-HCH | GC | <0.010 | <0.010 | <0.010 | <0.010 | 0.010 |
| Bifenazate-Bifenazate Diazene | GC | <0.010 | <0.010 | <0.010 | <0.010 | 0.010 |
| Bifenox | GC | <0.010 | <0.010 | <0.010 | <0.010 | 0.010 |
| Bifenthrin | GC | <0.025 | <0.025 | <0.025 | <0.025 | 0.025 |
| Bioallethrin | LC | <0.010 | <0.010 | <0.010 | <0.010 | 0.010 |
| Biphenyl | GC | <0.025 | <0.025 | <0.025 | <0.025 | 0.025 |
| Bitertanol | GC | <0.010 | <0.010 | <0.010 | <0.010 | 0.010 |
| Bixafen | LC | <0.010 | <0.010 | <0.010 | <0.010 | 0.010 |
| Boscalid | LC | <0.010 | <0.010 | <0.010 | <0.010 | 0.010 |
| Bromacil | LC | <0.010 | <0.010 | <0.010 | <0.010 | 0.010 |
| Bromophos-ethyl | GC | <0.010 | <0.010 | <0.010 | <0.010 | 0.010 |
| Bromophos-methyl | GC | <0.010 | <0.010 | <0.010 | <0.010 | 0.010 |
| Bromopropylat e | GC | <0.010 | <0.010 | <0.010 | <0.010 | 0.010 |
| Bromoxynil (SP) | LC | <0.010 | <0.010 | <0.010 | <0.010 | 0.010 |
| Bromuconazole | LC | <0.010 | <0.010 | <0.010 | <0.010 | 0.010 |
| BTS 44595 | LC | <0.010 | <0.010 | <0.010 | <0.010 | 0.010 |
| BTS 44596 | LC | <0.010 | <0.010 | <0.010 | <0.010 | 0.010 |
| Bupirimate (SP) | GC | <0.010 | <0.010 | <0.010 | <0.010 | 0.010 |
| Buprofezin | LC | <0.010 | <0.010 | <0.010 | <0.010 | 0.010 |
| Butachlor | LC | <0.010 | <0.010 | <0.010 | <0.010 | 0.010 |
| Butocarboxim | LC | <0.010 | <0.010 | <0.010 | <0.010 | 0.010 |
| Butoxicarboxim Sulfoxide | LC | <0.010 | <0.010 | <0.010 | <0.010 | 0.010 |

| | | | | | | |
|---|---|---|---|---|---|---|
| Butralin | LC | <0.010 | <0.010 | <0.010 | <0.010 | 0.010 |
| Buturon | LC | <0.010 | <0.010 | <0.010 | <0.010 | 0.010 |
| Cadusafos | LC | <0.010 | <0.010 | <0.010 | <0.010 | 0.010 |
| Captan | GC | <0.010 | <0.010 | <0.010 | <0.010 | 0.010 |
| Captan (sum) | GC | <0.010 | <0.010 | <0.010 | <0.010 | 0.010 |
| Carbaryl | LC | <0.010 | <0.010 | <0.010 | <0.010 | 0.010 |
| Carbendazim and Benomyl | LC | <0.010 | <0.010 | <0.010 | <0.010 | 0.010 |
| Carbetamide | LC | <0.010 | <0.010 | <0.010 | <0.010 | 0.010 |
| Carbofuran (SP/SQ) | LC | <0.010 | <0.010 | <0.010 | <0.010 | 0.010 |
| Carbophenothion | GC | <0.010 | <0.010 | <0.010 | <0.010 | 0.010 |
| Carboxin | LC | <0.010 | <0.010 | <0.010 | <0.010 | 0.010 |
| Carfentrazone-ethyl (SP) | LC | <0.010 | <0.010 | <0.010 | <0.010 | 0.010 |
| Chinomethionate | GC | <0.010 | <0.010 | <0.010 | <0.010 | 0.010 |
| Chlorantraniliprole | LC | <0.010 | <0.010 | <0.010 | <0.010 | 0.010 |
| Chlorbromuron | LC | <0.010 | <0.010 | <0.010 | <0.010 | 0.010 |
| Chlordane (Sum) | GC | <0.010 | <0.010 | <0.010 | <0.010 | 0.010 |
| Chlordane Cis | GC | <0.010 | <0.010 | <0.010 | <0.010 | 0.010 |
| Chlorfenapyr | GC | <0.010 | <0.010 | <0.010 | <0.010 | 0.010 |
| Chlorfenson | GC | <0.010 | <0.010 | <0.010 | <0.010 | 0.010 |
| Chlorfenvinphos | GC | <0.010 | <0.010 | <0.010 | <0.010 | 0.010 |
| Chlorfluazuron | LC | <0.010 | <0.010 | <0.010 | <0.010 | 0.010 |
| Chloridazon | LC | <0.010 | <0.010 | <0.010 | <0.010 | 0.010 |
| Chlormephos | GC | <0.010 | <0.010 | <0.010 | <0.010 | 0.010 |
| Chlorobenzilate +Chloropropylate | GC | <0.010 | <0.010 | <0.010 | <0.010 | 0.010 |
| Chlorothalonil | GC | <0.010 | <0.010 | <0.010 | <0.010 | 0.010 |
| Chlorotoluron | GC | <0.010 | <0.010 | <0.010 | <0.010 | 0.010 |
| Chloroxuron | LC | <0.010 | <0.010 | <0.010 | <0.010 | 0.010 |
| Chlorpropham (SP) | GC | <0.010 | <0.010 | <0.010 | <0.010 | 0.010 |
| Chlorpyrifos | GC | <0.010 | <0.010 | <0.010 | <0.010 | 0.010 |
| Chlorpyrifos-methyl | GC | <0.010 | <0.010 | <0.010 | <0.010 | 0.010 |
| Chlorsulfuron | LC | <0.010 | <0.010 | <0.010 | <0.010 | 0.010 |
| Chlorthal-dimethyl | GC | <0.010 | <0.010 | <0.010 | <0.010 | 0.010 |
| Chlorthion | GC | <0.010 | <0.010 | <0.010 | <0.010 | 0.010 |
| Chlorthiophos | LC | <0.010 | <0.010 | <0.010 | <0.010 | 0.010 |
| Chlozolinate | GC | <0.010 | <0.010 | <0.010 | <0.010 | 0.010 |
| Cinidon-ethyl | GC | <0.010 | <0.010 | <0.010 | <0.010 | 0.010 |
| Clethodim (SP) | LC | <0.010 | <0.010 | <0.010 | <0.010 | 0.010 |

| | | | | | | |
|---|---|---|---|---|---|---|
| Clethodim Sulfoxide | LC | <0.010 | <0.010 | <0.010 | <0.010 | 0.010 |
| Clofentezine | LC | <0.010 | <0.010 | <0.010 | <0.010 | 0.010 |
| Clomazone | LC | <0.010 | <0.010 | <0.010 | <0.010 | 0.010 |
| Clopyralid | LC | <0.150 | <0.150 | <0.150 | <0.150 | 0.150 |
| Clothianidin | LC | <0.010 | <0.010 | <0.010 | <0.010 | 0.010 |
| Coumaphos | LC | <0.010 | <0.010 | <0.010 | <0.010 | 0.010 |
| Crimidine | LC | <0.010 | <0.010 | <0.010 | <0.010 | 0.010 |
| Cyanazine | LC | <0.010 | <0.010 | <0.010 | <0.010 | 0.010 |
| Cyantraniliprole | LC | <0.010 | <0.010 | <0.010 | <0.010 | 0.010 |
| Cyazofamid | LC | <0.010 | <0.010 | <0.010 | <0.010 | 0.010 |
| Cyclanilide | LC | <0.010 | <0.010 | <0.010 | <0.010 | 0.010 |
| Cycloate | LC | <0.010 | <0.010 | <0.010 | <0.010 | 0.010 |
| Cycloxydim (SP) | LC | <0.010 | <0.010 | <0.010 | <0.010 | 0.010 |
| Cyenopyrafen | LC | <0.010 | <0.010 | <0.010 | <0.010 | 0.010 |
| Cyflufenamid | LC | <0.010 | <0.010 | <0.010 | <0.010 | 0.010 |
| Cyflumetofen | LC | <0.010 | <0.010 | <0.010 | <0.010 | 0.010 |
| Cyfluthrin | GC | <0.010 | <0.010 | <0.010 | <0.010 | 0.010 |
| Cyhalofop-butyl | LC | <0.010 | <0.010 | <0.010 | <0.010 | 0.010 |
| Cymoxanil | LC | <0.010 | <0.010 | <0.010 | <0.010 | 0.010 |
| Cyproconazole | GC | <0.010 | <0.010 | <0.010 | <0.010 | 0.010 |
| Cyprodinil | GC | <0.010 | <0.010 | <0.010 | <0.010 | 0.010 |
| Cyromazine | LC | <0.010 | <0.010 | <0.010 | <0.010 | 0.010 |
| DDD-pp+DDT-op | GC | <0.010 | <0.010 | <0.010 | <0.010 | 0.010 |
| DDT (Sum) | GC | <0.010 | <0.010 | <0.010 | <0.010 | 0.010 |
| DEET | GC | <0.025 | <0.025 | <0.025 | <0.025 | 0.025 |
| delta-HCH | GC | <0.025 | <0.010 | <0.010 | <0.010 | 0.010 |
| Deltamethrin | GC | <0.010 | <0.010 | <0.010 | <0.010 | 0.010 |
| Demeton S | LC | <0.010 | <0.010 | <0.010 | <0.010 | 0.010 |
| Demeton-S-methyl (SP) | LC | <0.010 | <0.010 | <0.010 | <0.010 | 0.010 |
| Demeton-S-methylsulfone | LC | <0.010 | <0.010 | <0.010 | <0.010 | 0.010 |
| Demeton-S-sulfoxide | LC | <0.010 | <0.010 | <0.010 | <0.010 | 0.010 |
| Desethyl atrazine | GC | <0.010 | <0.010 | <0.010 | <0.010 | 0.010 |
| Desmedipham | LC | <0.010 | <0.010 | <0.010 | <0.010 | 0.010 |
| Desmetryn | LC | <0.010 | <0.010 | <0.010 | <0.010 | 0.010 |
| Diafenthiuron | GC | <0.010 | <0.010 | <0.010 | <0.010 | 0.010 |
| Dialifos | LC | <0.010 | <0.010 | <0.010 | <0.010 | 0.010 |
| Diazinon | GC | <0.010 | <0.010 | <0.010 | <0.010 | 0.010 |
| Dichlobenil | GC | <0.010 | <0.010 | <0.010 | <0.010 | 0.010 |
| Dichlofenthion | GC | <0.010 | <0.010 | <0.010 | <0.010 | 0.010 |
| Dichlofluanid | LC | <0.010 | <0.010 | <0.010 | <0.010 | 0.010 |
| Dichlormid | LC | <0.010 | <0.010 | <0.010 | <0.010 | 0.010 |

| | | | | | | |
|---|---|---|---|---|---|---|
| Dichlorvos | LC | <0.010 | <0.010 | <0.010 | <0.010 | 0.010 |
| Diclobutrazol | GC | <0.010 | <0.010 | <0.010 | <0.010 | 0.010 |
| Diclofop (SP/SQ) | LC | <0.010 | <0.010 | <0.010 | <0.010 | 0.010 |
| Diclofop-methyl (SP/SQ) | LC | <0.010 | <0.010 | <0.010 | <0.010 | 0.010 |
| Diclofop (Sum) | LC | <0.010 | <0.010 | <0.010 | <0.010 | 0.010 |
| Dicloran | GC | <0.010 | <0.010 | <0.010 | <0.010 | 0.010 |
| Dicloroprop (added by Request) | LC | <0.010 | <0.010 | <0.010 | <0.010 | 0.010 |
| Dicofol (Sum) | GC | <0.010 | <0.010 | <0.010 | <0.010 | 0.010 |
| Dicofol o,p | GC | <0.010 | <0.010 | <0.010 | <0.010 | 0.010 |
| Dicofol p,p´ | GC | <0.010 | <0.010 | <0.010 | <0.010 | 0.010 |
| Dicrotophos | GC | <0.010 | <0.010 | <0.010 | <0.010 | 0.010 |
| Dieldrin (SP) | GC | <0.010 | <0.010 | <0.010 | <0.010 | 0.010 |
| Dieldrin (Sum) | GC | <0.010 | <0.010 | <0.010 | <0.010 | 0.010 |
| Diethofencarb | LC | <0.010 | <0.010 | <0.010 | <0.010 | 0.010 |
| Difenoconazole | GC | <0.010 | <0.010 | <0.010 | <0.010 | 0.010 |
| Diflubenzuron | LC | <0.010 | <0.010 | <0.010 | <0.010 | 0.010 |
| Diflufenican | GC | <0.010 | <0.010 | <0.010 | <0.010 | 0.010 |
| Dimefox | GC | <0.010 | <0.010 | <0.010 | <0.010 | 0.010 |
| Dimefuron | LC | <0.010 | <0.010 | <0.010 | <0.010 | 0.010 |
| Dimethachlor | LC | <0.010 | <0.010 | <0.010 | <0.010 | 0.010 |
| Dimethenamid-P | LC | <0.010 | <0.010 | <0.010 | <0.010 | 0.010 |
| Dimethoate | LC | <0.010 | <0.010 | <0.010 | <0.010 | 0.010 |
| Dimethoate (Sum) | LC | <0.010 | <0.010 | <0.010 | <0.010 | 0.010 |
| Dimethomorph | LC | <0.010 | <0.010 | <0.010 | <0.010 | 0.010 |
| Dimoxystrobin | GC | <0.010 | <0.010 | <0.010 | <0.010 | 0.010 |
| Diniconazole | GC | <0.010 | <0.010 | <0.010 | <0.010 | 0.010 |
| Dinobuton | GC | <0.010 | <0.010 | <0.010 | <0.010 | 0.010 |
| Dinotefuran | LC | <0.010 | <0.010 | <0.010 | <0.010 | 0.010 |
| Diphenylamine | GC | <0.010 | <0.010 | <0.010 | <0.010 | 0.010 |
| Disulfoton (SP) | GC | <0.010 | <0.010 | <0.010 | <0.010 | 0.010 |
| Disulfoton (Sum) | GC | <0.010 | <0.010 | <0.010 | <0.010 | 0.010 |
| Disulfoton-sulfone | GC | <0.010 | <0.010 | <0.010 | <0.010 | 0.010 |
| Disulfoton-sulfoxide | GC | <0.010 | <0.010 | <0.010 | <0.010 | 0.010 |
| Ditalimfos | GC | <0.010 | <0.010 | <0.010 | <0.010 | 0.010 |
| Diuron | LC | <0.010 | <0.010 | <0.010 | <0.010 | 0.010 |
| DMST | LC | <0.010 | <0.010 | <0.010 | <0.010 | 0.010 |
| DNOC | LC | <0.010 | <0.010 | <0.010 | <0.010 | 0.010 |
| Dodemorph | LC | <0.010 | <0.010 | <0.010 | <0.010 | 0.010 |
| Dodine | LC | <0.010 | <0.010 | <0.010 | <0.010 | 0.010 |
| Edifenphos | LC | <0.010 | <0.010 | <0.010 | <0.010 | 0.010 |

| | | | | | | |
|---|---|---|---|---|---|---|
| Emamectin B1a | LC | <0.010 | <0.010 | <0.010 | <0.010 | 0.010 |
| Endosulfan (A+B+Sulf) | GC | <0.010 | <0.010 | <0.010 | <0.010 | 0.010 |
| Endosulfan Sulfate | GC | <0.010 | <0.010 | <0.010 | <0.010 | 0.010 |
| Endrin | GC | <0.010 | <0.010 | <0.010 | <0.010 | 0.010 |
| EPN | GC | <0.010 | <0.010 | <0.010 | <0.010 | 0.010 |
| Epoxiconazole | LC | <0.010 | <0.010 | <0.010 | <0.010 | 0.010 |
| Epsilon-HCH | GC | <0.010 | <0.010 | <0.010 | <0.010 | 0.010 |
| EPTC | GC | <0.010 | <0.010 | <0.010 | <0.010 | 0.010 |
| Ethaboxam | LC | <0.010 | <0.010 | <0.010 | <0.010 | 0.010 |
| Ethalfluralin | GC | <0.010 | <0.010 | <0.010 | <0.010 | 0.010 |
| Ethiofencarb | LC | <0.010 | <0.010 | <0.010 | <0.010 | 0.010 |
| Ethiofencarb sulfone | LC | <0.010 | <0.010 | <0.010 | <0.010 | 0.010 |
| Ethiofencarb sulfoxide | LC | <0.010 | <0.010 | <0.010 | <0.010 | 0.010 |
| Ethion | GC | <0.010 | <0.010 | <0.010 | <0.010 | 0.010 |
| Ethiprole | LC | <0.010 | <0.010 | <0.010 | <0.010 | 0.010 |
| Ethirimol | LC | <0.010 | <0.010 | <0.010 | <0.010 | 0.010 |
| Ethofenprox | LC | <0.010 | <0.010 | <0.010 | <0.010 | 0.010 |
| Ethofumesate (SP) | GC | <0.010 | <0.010 | <0.010 | <0.010 | 0.010 |
| Ethoprophos | GC | <0.010 | <0.010 | <0.010 | <0.010 | 0.010 |
| Ethoxyquin | LC | <0.010 | <0.010 | <0.010 | <0.010 | 0.010 |
| Etoxazole | LC | <0.010 | <0.010 | <0.010 | <0.010 | 0.010 |
| Etridiazole | GC | <0.010 | <0.010 | <0.010 | <0.010 | 0.010 |
| Etrimfos | GC | <0.010 | <0.010 | <0.010 | <0.010 | 0.010 |
| Famoxadone | LC | <0.010 | <0.010 | <0.010 | <0.010 | 0.010 |
| Fenamidone | LC | <0.010 | <0.010 | <0.010 | <0.010 | 0.010 |
| Fenamiphos (SP) | LC | <0.010 | <0.010 | <0.010 | <0.010 | 0.010 |
| Fenamiphos (Sum) | LC | <0.010 | <0.010 | <0.010 | <0.010 | 0.010 |
| Fenamiphos sulfone | LC | <0.010 | <0.010 | <0.010 | <0.010 | 0.010 |
| Fenamiphos sulfoxide | LC | <0.010 | <0.010 | <0.010 | <0.010 | 0.010 |
| Fenarimol | GC | <0.010 | <0.010 | <0.010 | <0.010 | 0.010 |
| Fenazaquin | GC | <0.010 | <0.010 | <0.010 | <0.010 | 0.010 |
| Fenchlorphos | GC | <0.010 | <0.010 | <0.010 | <0.010 | 0.010 |
| Fenchlorphos (Sum) | GC | <0.010 | <0.010 | <0.010 | <0.010 | 0.010 |
| Fenchlorphos Oxon | GC | <0.010 | <0.010 | <0.010 | <0.010 | 0.010 |
| Fenhexamid | LC | <0.010 | <0.010 | <0.010 | <0.010 | 0.010 |

| | | | | | | |
|---|---|---|---|---|---|---|
| Fenitrothion | GC | <0.010 | <0.010 | <0.010 | <0.010 | 0.010 |
| Fenobucarb | LC | <0.010 | <0.010 | <0.010 | <0.010 | 0.010 |
| Fenoxycarb | LC | <0.010 | <0.010 | <0.010 | <0.010 | 0.010 |
| Fenpiclonil | LC | <0.010 | <0.010 | <0.010 | <0.010 | 0.010 |
| Fenpropathrin | GC | <0.010 | <0.010 | <0.010 | <0.010 | 0.010 |
| Fenpropidin | LC | <0.010 | <0.010 | <0.010 | <0.010 | 0.010 |
| Fenpropimorph | LC | <0.010 | <0.010 | <0.010 | <0.010 | 0.010 |
| Fenpyrazamine | LC | <0.010 | <0.010 | <0.010 | <0.010 | 0.010 |
| Fenpyroximate | LC | <0.010 | <0.010 | <0.010 | <0.010 | 0.010 |
| Fenson | GC | <0.010 | <0.010 | <0.010 | <0.010 | 0.010 |
| Fensulfothion | LC | <0.010 | <0.010 | <0.010 | <0.010 | 0.010 |
| Fensulfothion Oxon | LC | <0.010 | <0.010 | <0.010 | <0.010 | 0.010 |
| Fensulfothion Oxon Sulfone | LC | <0.010 | <0.010 | <0.010 | <0.010 | 0.010 |
| Fensulfothion Sulfone | LC | <0.010 | <0.010 | <0.010 | <0.010 | 0.010 |
| Fenthion (SP) | GC | <0.010 | <0.010 | <0.010 | <0.010 | 0.010 |
| Fenthion (Sum) | LC | <0.010 | <0.010 | <0.010 | <0.010 | 0.010 |
| Fenthion Oxon | GC | <0.010 | <0.010 | <0.010 | <0.010 | 0.010 |
| Fenthion Oxon Sulfone | LC | <0.010 | <0.010 | <0.010 | <0.010 | 0.010 |
| Fenthion Oxon Sulfoxide | LC | <0.010 | <0.010 | <0.010 | <0.010 | 0.010 |
| Fenthion-sulfone | LC | <0.010 | <0.010 | <0.010 | <0.010 | 0.010 |
| Fenthion-sulfoxide | LC | <0.010 | <0.010 | <0.010 | <0.010 | 0.010 |
| Fentin (SP/SQ) | LC | <0.010 | <0.010 | <0.010 | <0.010 | 0.010 |
| Fenuron | LC | <0.010 | <0.010 | <0.010 | <0.010 | 0.010 |
| Fenvalerate | GC | <0.010 | <0.010 | <0.010 | <0.010 | 0.010 |
| Fipronil (SP) | LC | <0.012 | <0.012 | <0.012 | <0.012 | 0.012 |
| Fipronil (Sum) | LC | <0.012 | <0.012 | <0.012 | <0.012 | 0.012 |
| Fipronil Sulfide | LC | <0.012 | <0.012 | <0.012 | <0.012 | 0.012 |
| Fipronil Sulfone | LC | <0.012 | <0.012 | <0.012 | <0.012 | 0.012 |
| Flamprop | LC | <0.010 | <0.010 | <0.010 | <0.010 | 0.010 |
| Flazasulfuron | LC | <0.010 | <0.010 | <0.010 | <0.010 | 0.010 |
| Flonicamid (SP) | LC | <0.010 | <0.010 | <0.010 | <0.010 | 0.010 |
| Flonicamid (Sum) | LC | <0.010 | <0.010 | <0.010 | <0.010 | 0.010 |
| Florasulam | LC | <0.010 | <0.010 | <0.010 | <0.010 | 0.010 |
| Fluazifop Methyl (SP) | LC | <0.010 | <0.010 | <0.010 | <0.010 | 0.010 |
| Fluazifop-P (SP) | LC | <0.010 | <0.010 | <0.010 | <0.010 | 0.010 |

| | | | | | | |
|---|---|---|---|---|---|---|
| Fluazifop-P-butyl (SP) | LC | <0.010 | <0.010 | <0.010 | <0.010 | 0.010 |
| Fluazinam | LC | <0.010 | <0.010 | <0.010 | <0.010 | 0.010 |
| Flubendiamide | LC | <0.010 | <0.010 | <0.010 | <0.010 | 0.010 |
| Flucythrinate | GC | <0.010 | <0.010 | <0.010 | <0.010 | 0.010 |
| Fludioxonil | LC | <0.010 | <0.010 | <0.010 | <0.010 | 0.010 |
| Flufenacet | LC | <0.010 | <0.010 | <0.010 | <0.010 | 0.010 |
| Flufenacet (Sum) | LC | <0.010 | <0.010 | <0.010 | <0.010 | 0.010 |
| Flufenacet ESA | LC | <0.010 | <0.010 | <0.010 | <0.010 | 0.010 |
| Flufenacet OA | LC | <0.010 | <0.010 | <0.010 | <0.010 | 0.010 |
| Flufenoxuron | LC | <0.010 | <0.010 | <0.010 | <0.010 | 0.010 |
| Flumetralin | GC | <0.010 | <0.010 | <0.010 | <0.010 | 0.010 |
| Flumioxazin | LC | <0.010 | <0.010 | <0.010 | <0.010 | 0.010 |
| Fluometuron | LC | <0.010 | <0.010 | <0.010 | <0.010 | 0.010 |
| Fluopicolide | GC | <0.010 | <0.010 | <0.010 | <0.010 | 0.010 |
| Fluopyram | GC | <0.010 | <0.010 | <0.010 | <0.010 | 0.010 |
| Fluotrimazole | GC | <0.010 | <0.010 | <0.010 | <0.010 | 0.010 |
| Fluoxastrobin | LC | <0.010 | <0.010 | <0.010 | <0.010 | 0.010 |
| Flupyradifurone | LC | <0.010 | <0.010 | <0.010 | <0.010 | 0.010 |
| Fluquinconazol | LC | <0.010 | <0.010 | <0.010 | <0.010 | 0.010 |
| Fluroxypyr (SP) | LC | <0.010 | <0.010 | <0.010 | <0.010 | 0.010 |
| Fluroxypyr-meptyl | LC | <0.010 | <0.010 | <0.010 | <0.010 | 0.010 |
| Flurtamone | GC | <0.010 | <0.010 | <0.010 | <0.010 | 0.010 |
| Flusilazole | LC | <0.010 | <0.010 | <0.010 | <0.010 | 0.010 |
| Flutolanil | LC | <0.010 | <0.010 | <0.010 | <0.010 | 0.010 |
| Flutriafol | LC | <0.010 | <0.010 | <0.010 | <0.010 | 0.010 |
| Fluxapyroxad | LC | <0.010 | <0.010 | <0.010 | <0.010 | 0.010 |
| Fluvalinate | GC | <0.010 | <0.010 | <0.010 | <0.010 | 0.010 |
| Folpet | GC | <0.010 | <0.010 | <0.010 | <0.010 | 0.010 |
| Folpet (Sum) | GC | <0.150 | <0.150 | <0.150 | <0.150 | 0.150 |
| Fonofos | GC | <0.010 | <0.010 | <0.010 | <0.010 | 0.010 |
| Foramsulfuron | LC | <0.010 | <0.010 | <0.010 | <0.010 | 0.010 |
| Forchlorfenuron | LC | <0.010 | <0.010 | <0.010 | <0.010 | 0.010 |
| Formetanate (SP) | LC | <0.010 | <0.010 | <0.010 | <0.010 | 0.010 |
| Formothion | LC | <0.010 | <0.010 | <0.010 | <0.010 | 0.010 |
| Fosthiazate | LC | <0.010 | <0.010 | <0.010 | <0.010 | 0.010 |
| Fuberidazole | LC | <0.010 | <0.010 | <0.010 | <0.010 | 0.010 |
| Furalaxyl | GC | <0.010 | <0.010 | <0.010 | <0.010 | 0.010 |
| Halosulfuron-methyl | LC | <0.010 | <0.010 | <0.010 | <0.010 | 0.010 |
| ~~Haloxyfop~~ | LC | <0.010 | <0.010 | <0.010 | <0.010 | 0.010 |

| | | | | | | |
|---|---|---|---|---|---|---|
| Haloxyfop (Sum) | LC | <0.010 | <0.010 | <0.010 | <0.010 | 0.010 |
| Haloxyfop-2-ethoxyethyl | LC | <0.010 | <0.010 | <0.010 | <0.010 | 0.010 |
| Haloxyfop-methyl (SP) | LC | <0.010 | <0.010 | <0.010 | <0.010 | 0.010 |
| Haloxyfop-R (SP) | LC | <0.010 | <0.010 | <0.010 | <0.010 | 0.010 |
| Heptachlor (SP) | GC | <0.010 | <0.010 | <0.010 | <0.010 | 0.010 |
| Heptachlor (Sum) | GC | <0.010 | <0.010 | <0.010 | <0.010 | 0.010 |
| Heptachlor Epoxide A | GC | <0.010 | <0.010 | <0.010 | <0.010 | 0.010 |
| Heptachlor Epoxide B | GC | <0.010 | <0.010 | <0.010 | <0.010 | 0.010 |
| Heptenophos | GC | <0.010 | <0.010 | <0.010 | <0.010 | 0.010 |
| Hexachlorobenzene | GC | <0.010 | <0.010 | <0.010 | <0.010 | 0.010 |
| Hexaclorobutadiene | GC | <0.010 | <0.010 | <0.010 | <0.010 | 0.010 |
| Hexaconazole | GC | <0.010 | <0.010 | <0.010 | <0.010 | 0.010 |
| Hexaflumuron | LC | <0.010 | <0.010 | <0.010 | <0.010 | 0.010 |
| Hexazinone | LC | <0.010 | <0.010 | <0.010 | <0.010 | 0.010 |
| Hexythiazox | LC | <0.010 | <0.010 | <0.010 | <0.010 | 0.010 |
| Imazalil | LC | <0.010 | <0.010 | <0.010 | <0.010 | 0.010 |
| Imazapic (Added by Request) | LC | <0.010 | <0.010 | <0.010 | <0.010 | 0.010 |
| Imazapyr (added by request) | LC | <0.010 | <0.010 | <0.010 | <0.010 | 0.010 |
| Imidacloprid (SP) | LC | <0.010 | <0.010 | <0.010 | <0.010 | 0.010 |
| Indaziflam | LC | <0.010 | <0.010 | <0.010 | <0.010 | 0.010 |
| Indoxacarb | LC | <0.010 | <0.010 | <0.010 | <0.010 | 0.010 |
| Iodofenphos | GC | <0.010 | <0.010 | <0.010 | <0.010 | 0.010 |
| Iodosulfuron-methyl (SP) | LC | <0.010 | <0.010 | <0.010 | <0.010 | 0.010 |
| Ioxynil (SP) | LC | <0.010 | <0.010 | <0.010 | <0.010 | 0.010 |
| Iprobenfos | GC | <0.010 | <0.010 | <0.010 | <0.010 | 0.010 |
| Iprodione | GC | <0.010 | <0.010 | <0.010 | <0.010 | 0.010 |
| Iprovalicarb | GC | <0.010 | <0.010 | <0.010 | <0.010 | 0.010 |
| Isazofos | GC | <0.010 | <0.010 | <0.010 | <0.010 | 0.010 |
| Isocarbophos | LC | | | <0.010 | <0.010 | 0.010 |
| Isofenphos | GC | <0.010 | <0.010 | <0.010 | <0.010 | 0.010 |
| Isophenfos-methyl | GC | <0.010 | <0.010 | <0.010 | <0.010 | 0.010 |
| Isoprocarb | LC | <0.010 | <0.010 | <0.010 | <0.010 | 0.010 |

| | | | | | | |
|---|---|---|---|---|---|---|
| Isoprocarbphos | LC | <0.010 | <0.010 | <0.010 | <0.010 | 0.010 |
| Isoprothiolane | LC | <0.010 | <0.010 | <0.010 | <0.010 | 0.010 |
| Isoproturon | LC | <0.010 | <0.010 | <0.010 | <0.010 | 0.010 |
| Isopyrazam | LC | <0.010 | <0.010 | <0.010 | <0.010 | 0.010 |
| Isoxaben | LC | <0.010 | <0.010 | <0.010 | <0.010 | 0.010 |
| Isoxathion | LC | <0.010 | <0.010 | <0.010 | <0.010 | 0.010 |
| Isoxaben | LC | <0.010 | <0.010 | <0.010 | <0.010 | 0.010 |
| Ivermectin | LC | <0.010 | <0.010 | <0.010 | <0.010 | 0.010 |
| Kresoxim-methyl | GC | <0.010 | <0.010 | <0.010 | <0.010 | 0.010 |
| Lambda-Cyhalothrin | GC | <0.010 | <0.010 | <0.010 | <0.010 | 0.010 |
| Lenacile | LC | <0.010 | <0.010 | <0.010 | <0.010 | 0.010 |
| Lindane | GC | <0.010 | <0.010 | <0.010 | <0.010 | 0.010 |
| Linuron | LC | <0.010 | <0.010 | <0.010 | <0.010 | 0.010 |
| Lufenuron | LC | <0.010 | <0.010 | <0.010 | <0.010 | 0.010 |
| Malaoxon | GC | <0.010 | <0.010 | <0.010 | <0.010 | 0.010 |
| Malathion (SP) | GC | <0.010 | <0.010 | <0.010 | <0.010 | 0.010 |
| Malathion (Sum) | GC | <0.010 | <0.010 | <0.010 | <0.010 | 0.010 |
| Mandipropamid | LC | <0.010 | <0.010 | <0.010 | <0.010 | 0.010 |
| Matrine | LC | <0.010 | <0.010 | <0.010 | <0.010 | 0.010 |
| MCPA | LC | <0.010 | <0.010 | <0.010 | <0.010 | 0.010 |
| Mecarbam | LC | <0.010 | <0.010 | <0.010 | <0.010 | 0.010 |
| Mefenpyr Diethyl | GC | <0.010 | <0.010 | <0.010 | <0.010 | 0.010 |
| Mepanipyrim | LC | <0.010 | <0.010 | <0.010 | <0.010 | 0.010 |
| Mepronil | GC | <0.010 | <0.010 | <0.010 | <0.010 | 0.010 |
| Meptyldinocap | LC | <0.010 | <0.010 | <0.010 | <0.010 | 0.010 |
| Mesosulfuron-methyl | LC | <0.010 | <0.010 | <0.010 | <0.010 | 0.010 |
| Mesotrione | LC | <0.010 | <0.010 | <0.010 | <0.010 | 0.010 |
| Metaflumizone | LC | <0.010 | <0.010 | <0.010 | <0.010 | 0.010 |
| Metalaxyl-M | GC | <0.010 | <0.010 | <0.010 | <0.010 | 0.010 |
| Metamitron | LC | <0.010 | <0.010 | <0.010 | <0.010 | 0.010 |
| Metazachlor (SP) | LC | <0.010 | <0.010 | <0.010 | <0.010 | 0.010 |
| Metconazole | LC | <0.010 | <0.010 | <0.010 | <0.010 | 0.010 |
| Methabenzthiazuron | LC | <0.010 | <0.010 | <0.010 | <0.010 | 0.010 |
| Methacrifos | GC | <0.010 | <0.010 | <0.010 | <0.010 | 0.010 |
| Methamidophos | LC | <0.010 | <0.010 | <0.010 | <0.010 | 0.010 |
| Methidathion | GC | <0.010 | <0.010 | <0.010 | <0.010 | 0.010 |
| Methiocarb (SP) | LC | <0.010 | <0.010 | <0.010 | <0.010 | 0.010 |

| | | | | | | |
|---|---|---|---|---|---|---|
| Methiocarb (Sum) | LC | <0.010 | <0.010 | <0.010 | <0.010 | 0.010 |
| Methiocarb sulfone | LC | <0.010 | <0.010 | <0.010 | <0.010 | 0.010 |
| Methiocarb sulfoxide | LC | <0.010 | <0.010 | <0.010 | <0.010 | 0.010 |
| Methomyl | LC | <0.010 | <0.010 | <0.010 | <0.010 | 0.010 |
| Methomyl (Sum) | LC | <0.010 | <0.010 | <0.010 | <0.010 | 0.010 |
| Methoprotryne | LC | <0.010 | <0.010 | <0.010 | <0.010 | 0.010 |
| Methoxychlor | GC | <0.010 | <0.010 | <0.010 | <0.010 | 0.010 |
| Methoxyfenozide | LC | <0.010 | <0.010 | <0.010 | <0.010 | 0.010 |
| Metobromuron | LC | <0.010 | <0.010 | <0.010 | <0.010 | 0.010 |
| Metolachlor and S-Metolachlor | LC | <0.010 | <0.010 | <0.010 | <0.010 | 0.010 |
| Metolcarb | LC | <0.010 | <0.010 | <0.010 | <0.010 | 0.010 |
| Metoxuron | LC | <0.010 | <0.010 | <0.010 | <0.010 | 0.010 |
| Metrafenone | LC | <0.010 | <0.010 | <0.010 | <0.010 | 0.010 |
| Metribuzin | GC | <0.010 | <0.010 | <0.010 | <0.010 | 0.010 |
| Metsulphuron-methyl | LC | <0.010 | <0.010 | <0.010 | <0.010 | 0.010 |
| Mevinphos | GC | <0.010 | <0.010 | <0.010 | <0.010 | 0.010 |
| Milbemectin ( SQ Sum) | LC | <0.010 | <0.010 | <0.010 | <0.010 | 0.010 |
| Milbemycin A3 (SQ) | LC | <0.010 | <0.010 | <0.010 | <0.010 | 0.010 |
| Milbemycin A4 (SQ) | LC | <0.010 | <0.010 | <0.010 | <0.010 | 0.010 |
| Mirex | GC | <0.010 | <0.010 | <0.010 | <0.010 | 0.010 |
| Molinate | GC | <0.010 | <0.010 | <0.010 | <0.010 | 0.010 |
| Monocrotophos | LC | <0.010 | <0.010 | <0.010 | <0.010 | 0.010 |
| Monolinuron | LC | <0.010 | <0.010 | <0.010 | <0.010 | 0.010 |
| Monuron | LC | <0.010 | <0.010 | <0.010 | <0.010 | 0.010 |
| Myclobutanil | GC | <0.010 | <0.010 | <0.010 | <0.010 | 0.010 |
| Naled | GC | <0.010 | <0.010 | <0.010 | <0.010 | 0.010 |
| Naled Sum | GC | <0.010 | <0.010 | <0.010 | <0.010 | 0.010 |
| Napropamide | GC | <0.010 | <0.010 | <0.010 | <0.010 | 0.010 |
| Neburon | LC | <0.010 | <0.010 | <0.010 | <0.010 | 0.010 |
| Nicosulfuron | LC | <0.010 | <0.010 | <0.010 | <0.010 | 0.010 |
| Nitenpyram | LC | <0.010 | <0.010 | <0.010 | <0.010 | 0.010 |
| Nitrofen | GC | <0.010 | <0.010 | <0.010 | <0.010 | 0.010 |
| Nitrothal Isopropyl | GC | <0.010 | <0.010 | <0.010 | <0.010 | 0.010 |
| Norflurazon | LC | <0.010 | <0.010 | <0.010 | <0.010 | 0.010 |
| Novaluron | LC | <0.010 | <0.010 | <0.010 | <0.010 | 0.010 |
| Nuarimol | GC | <0.010 | <0.010 | <0.010 | <0.010 | 0.010 |

| | | | | | | |
|---|---|---|---|---|---|---|
| O, p-DDD | GC | <0.010 | <0.010 | <0.010 | <0.010 | 0.010 |
| O,p-DDE | GC | <0.010 | <0.010 | <0.010 | <0.010 | 0.010 |
| Ofurace | GC | <0.010 | <0.010 | <0.010 | <0.010 | 0.010 |
| Omethoate (SP) | LC | <0.010 | <0.010 | <0.010 | <0.010 | 0.010 |
| Oryzalin | LC | <0.010 | <0.010 | <0.010 | <0.010 | 0.010 |
| Oxadiargyl | LC | <0.010 | <0.010 | <0.010 | <0.010 | 0.010 |
| Oxadiazon | LC | <0.010 | <0.010 | <0.010 | <0.010 | 0.010 |
| Oxadixyl | GC | <0.010 | <0.010 | <0.010 | <0.010 | 0.010 |
| Oxamyl | LC | <0.010 | <0.010 | <0.010 | <0.010 | 0.010 |
| Oxasulfuron | LC | <0.010 | <0.010 | <0.010 | <0.010 | 0.010 |
| Oxathiapiprolin | LC | <0.010 | <0.010 | <0.010 | <0.010 | 0.010 |
| Oxycarboxin | LC | <0.010 | <0.010 | <0.010 | <0.010 | 0.010 |
| Oxychlordan | GC | <0.010 | <0.010 | <0.010 | <0.010 | 0.010 |
| Oxyfluorfen | GC | <0.010 | <0.010 | <0.010 | <0.010 | 0.010 |
| P,p- DDT | GC | <0.010 | <0.010 | <0.010 | <0.010 | 0.010 |
| P,p-DDE | GC | <0.010 | <0.010 | <0.010 | <0.010 | 0.010 |
| Paclobutrazol | LC | <0.010 | <0.010 | <0.010 | <0.010 | 0.010 |
| Paraoxon-ethyl | GC | <0.010 | <0.010 | <0.010 | <0.010 | 0.010 |
| Paraoxon-methyl | GC | <0.010 | <0.010 | <0.010 | <0.010 | 0.010 |
| Parathion-ethyl | GC | <0.010 | <0.010 | <0.010 | <0.010 | 0.010 |
| Parathion-ethyl (sum) | GC | <0.010 | <0.010 | <0.010 | <0.010 | 0.010 |
| Parathion-methyl (SP) | GC | <0.010 | <0.010 | <0.010 | <0.010 | 0.010 |
| Parathion-methyl (Sum) | GC | <0.010 | <0.010 | <0.010 | <0.010 | 0.010 |
| Penconazole | GC | <0.010 | <0.010 | <0.010 | <0.010 | 0.010 |
| Pencycuron | LC | <0.010 | <0.010 | <0.010 | <0.010 | 0.010 |
| Pendimethalin | GC | <0.010 | <0.010 | <0.010 | <0.010 | 0.010 |
| Pentachloroaniline | GC | <0.010 | <0.010 | <0.010 | <0.010 | 0.010 |
| Pentachloroanisole | GC | <0.010 | <0.010 | <0.010 | <0.010 | 0.010 |
| Pentachlorobenzene | GC | <0.010 | <0.010 | <0.010 | <0.010 | 0.010 |
| Pentachlorobenzonitrile | GC | <0.010 | <0.010 | <0.010 | <0.010 | 0.010 |
| Pentachlorophenol | GC | <0.010 | <0.010 | <0.010 | <0.010 | 0.010 |
| Penthiopyrad | LC | <0.010 | <0.010 | <0.010 | <0.010 | 0.010 |
| Permethrin | GC | <0.010 | <0.010 | <0.010 | <0.010 | 0.010 |
| Phenmedipham | LC | <0.010 | <0.010 | <0.010 | <0.010 | 0.010 |
| Phenthoate | GC | <0.010 | <0.010 | <0.010 | <0.010 | 0.010 |
| Phorate | GC | <0.010 | <0.010 | <0.010 | <0.010 | 0.010 |
| Phorate (Sum) | LC | <0.010 | <0.010 | <0.010 | <0.010 | 0.010 |

| | | | | | | |
|---|---|---|---|---|---|---|
| Phorate Oxon | LC | <0.010 | <0.010 | <0.010 | <0.010 | 0.010 |
| Phorate Oxon Sulfone | LC | <0.010 | <0.010 | <0.010 | <0.010 | 0.010 |
| Phorate Oxon Sulfoxide | LC | <0.010 | <0.010 | <0.010 | <0.010 | 0.010 |
| Phorate sulfone | LC | <0.010 | <0.010 | <0.010 | <0.010 | 0.010 |
| Phorate sulfoxide | LC | <0.010 | <0.010 | <0.010 | <0.010 | 0.010 |
| Phosalone | GC | <0.010 | <0.010 | <0.010 | <0.010 | 0.010 |
| Phosmet (SP) | LC | <0.010 | <0.010 | <0.010 | <0.010 | 0.010 |
| Phosmet (Sum) | LC | <0.010 | <0.010 | <0.010 | <0.010 | 0.010 |
| Phosmet-oxon | LC | <0.010 | <0.010 | <0.010 | <0.010 | 0.010 |
| Phosphamidon | LC | <0.010 | <0.010 | <0.010 | <0.010 | 0.010 |
| Phoxim | LC | <0.010 | <0.010 | <0.010 | <0.010 | 0.010 |
| Phthalimide (Folpet) | GC | <0.150 | <0.150 | <0.150 | <0.150 | 0.150 |
| Picolinafen | LC | <0.010 | <0.010 | <0.010 | <0.010 | 0.010 |
| Picoxystrobin | LC | <0.010 | <0.010 | <0.010 | <0.010 | 0.010 |
| Pinoxaden | LC | <0.010 | <0.010 | <0.010 | <0.010 | 0.010 |
| Piperonyl butoxide | GC | <0.010 | <0.010 | <0.010 | <0.010 | 0.010 |
| Pirimicarb | LC | <0.010 | <0.010 | <0.010 | <0.010 | 0.010 |
| Pirimicarb Desmethyl | LC | <0.010 | <0.010 | <0.010 | <0.010 | 0.010 |
| Pirimicarb Desmethyl Formamide | LC | <0.010 | <0.010 | <0.010 | <0.010 | 0.010 |
| Pirimiphos-ethyl | GC | <0.010 | <0.010 | <0.010 | <0.010 | 0.010 |
| Pirimiphos-methyl | GC | <0.010 | <0.010 | <0.010 | <0.010 | 0.010 |
| Prochloraz (SP) | LC | <0.010 | <0.010 | <0.010 | <0.010 | 0.010 |
| Prochloraz (Sum) | LC | <0.010 | <0.010 | <0.010 | <0.010 | 0.010 |
| Procymidone | GC | <0.010 | <0.010 | <0.010 | <0.010 | 0.010 |
| Profenofos | GC | <0.010 | <0.010 | <0.010 | <0.010 | 0.010 |
| Profluralin | GC | <0.010 | <0.010 | <0.010 | <0.010 | 0.010 |
| Promecarb | LC | <0.010 | <0.010 | <0.010 | <0.010 | 0.010 |
| Prometryn | GC | <0.010 | <0.010 | <0.010 | <0.010 | 0.010 |
| Propachlor | LC | <0.010 | <0.010 | <0.010 | <0.010 | 0.010 |
| Propamocarb (SP) | LC | <0.010 | <0.010 | <0.010 | <0.010 | 0.010 |
| Propanil | LC | <0.010 | <0.010 | <0.010 | <0.010 | 0.010 |
| Propaquizafop | LC | <0.010 | <0.010 | <0.010 | <0.010 | 0.010 |
| Propargite | LC | <0.010 | <0.010 | <0.010 | <0.010 | 0.010 |
| Propazine | GC | <0.010 | <0.010 | <0.010 | <0.010 | 0.010 |

| | | | | | | |
|---|---|---|---|---|---|---|
| Propetamphos | GC | <0.010 | <0.010 | <0.010 | <0.010 | 0.010 |
| Propham | LC | <0.010 | <0.010 | <0.010 | <0.010 | 0.010 |
| Propiconazole | LC | <0.010 | <0.010 | <0.010 | <0.010 | 0.010 |
| Propoxur | LC | <0.025 | <0.025 | <0.025 | <0.010 | 0.010 |
| Propyzamide | GC | <0.010 | <0.010 | <0.010 | <0.010 | 0.010 |
| Proquinazid | LC | <0.010 | <0.010 | <0.010 | <0.010 | 0.010 |
| Prosulfocarb | LC | <0.010 | <0.010 | <0.010 | <0.010 | 0.010 |
| Prosulfuron | LC | <0.010 | <0.010 | <0.010 | <0.010 | 0.010 |
| Prothioconazole | LC | <0.010 | <0.010 | <0.010 | <0.010 | 0.010 |
| Prothiofos | GC | <0.010 | <0.010 | <0.010 | <0.010 | 0.010 |
| Pydiflumetofen | LC | <0.010 | <0.010 | <0.010 | <0.010 | 0.010 |
| Pymetrozine | LC | <0.010 | <0.010 | <0.010 | <0.010 | 0.010 |
| Pyracarbolid | LC | <0.010 | <0.010 | <0.010 | <0.010 | 0.010 |
| Pyraclostrobin | LC | <0.010 | <0.010 | <0.010 | <0.010 | 0.010 |
| Pyraflufen | LC | <0.010 | <0.010 | <0.010 | <0.010 | 0.010 |
| Pyraflufen-ethyl (SP) | LC | <0.010 | <0.010 | <0.010 | <0.010 | 0.010 |
| Pyraflufen-ethyl (Sum) | LC | <0.010 | <0.010 | <0.010 | <0.010 | 0.010 |
| Pyrazophos | GC | <0.010 | <0.010 | <0.010 | <0.010 | 0.010 |
| Pyridaben | GC | <0.010 | <0.010 | <0.010 | <0.010 | 0.010 |
| Pyridalyl | LC | <0.010 | <0.010 | <0.010 | <0.010 | 0.010 |
| Pyridaphenthion | GC | <0.010 | <0.010 | <0.010 | <0.010 | 0.010 |
| Pyridate (SP) | LC | <0.010 | <0.010 | <0.010 | <0.010 | 0.010 |
| Pyrifenox | GC | <0.010 | <0.010 | <0.010 | <0.010 | 0.010 |
| Pyrimethanil | GC | <0.010 | <0.010 | <0.010 | <0.010 | 0.010 |
| Pyriproxyfen | GC | <0.010 | <0.010 | <0.010 | <0.010 | 0.010 |
| Quinalphos | GC | <0.010 | <0.010 | <0.010 | <0.010 | 0.010 |
| Quinclorac | LC | <0.010 | <0.010 | <0.010 | <0.010 | 0.010 |
| Quinoxyfen | LC | <0.010 | <0.010 | <0.010 | <0.010 | 0.010 |
| Quintozene | GC | <0.010 | <0.010 | <0.010 | <0.010 | 0.010 |
| Quintozene (Sum) | GC | <0.010 | <0.010 | <0.010 | <0.010 | 0.010 |
| Quizalofop-ethyl (SP) | LC | <0.010 | <0.010 | <0.010 | <0.010 | 0.010 |
| Rimsulfuron | LC | <0.010 | <0.010 | <0.010 | <0.010 | 0.010 |
| Rotenone | LC | <0.010 | <0.010 | <0.010 | <0.010 | 0.010 |
| Saflufenacil (SP) | LC | <0.010 | <0.010 | <0.010 | <0.010 | 0.010 |
| Sebuthylazine | LC | <0.010 | <0.010 | <0.010 | <0.010 | 0.010 |
| Sethoxydim | LC | <0.010 | <0.010 | <0.010 | <0.010 | 0.010 |
| Silthiofam | GC | <0.010 | <0.010 | <0.010 | <0.010 | 0.010 |
| Simazine | GC | <0.010 | <0.010 | <0.010 | <0.010 | 0.010 |
| Spinetoram | LC | <0.010 | <0.010 | <0.010 | <0.010 | 0.010 |
| Spinosad | LC | <0.010 | <0.010 | <0.010 | <0.010 | 0.010 |

| | | | | | | |
|---|---|---|---|---|---|---|
| Spirodiclofen | LC | <0.010 | <0.010 | <0.010 | <0.010 | 0.010 |
| Spiromesifen | LC | <0.010 | <0.010 | <0.010 | <0.010 | 0.010 |
| Spirotetramat (SP) | LC | <0.010 | <0.010 | <0.010 | <0.010 | 0.010 |
| Spirotetramat-keto-hydroxy | LC | <0.010 | <0.010 | <0.010 | <0.010 | 0.010 |
| Spirotetramat-enol-glucoside | LC | <0.010 | <0.010 | <0.010 | <0.010 | 0.010 |
| Spirotetramat-enol | LC | <0.010 | <0.010 | <0.010 | <0.010 | 0.010 |
| Spirotetramat-mono-hydroxy | LC | <0.010 | <0.010 | <0.010 | <0.010 | 0.010 |
| Spiroxamine | LC | <0.010 | <0.010 | <0.010 | <0.010 | 0.010 |
| Sulcotrione | LC | <0.010 | <0.010 | <0.010 | <0.010 | 0.010 |
| Sulfosulfuron | LC | <0.010 | <0.010 | <0.010 | <0.010 | 0.010 |
| Sulfotep | LC | <0.010 | <0.010 | <0.010 | <0.010 | 0.010 |
| Sulfoxaflor | LC | <0.010 | <0.010 | <0.010 | <0.010 | 0.010 |
| Tebuconazole | GC | <0.010 | <0.010 | <0.010 | <0.010 | 0.010 |
| Tebufenozide | LC | <0.010 | <0.010 | <0.010 | <0.010 | 0.010 |
| Tebufenpyrad | GC | <0.010 | <0.010 | <0.010 | <0.010 | 0.010 |
| Tecnazene | GC | <0.010 | <0.010 | <0.010 | <0.010 | 0.010 |
| Teflubenzuron | LC | <0.010 | <0.010 | <0.010 | <0.010 | 0.010 |
| Tefluthrin | GC | <0.010 | <0.010 | <0.010 | <0.010 | 0.010 |
| Tepraloxydim (SP) | LC | <0.010 | <0.010 | <0.010 | <0.010 | 0.010 |
| Terbacil | GC | <0.010 | <0.010 | <0.010 | <0.010 | 0.010 |
| Terbufos | LC | <0.010 | <0.010 | <0.010 | <0.010 | 0.010 |
| Terbufos (Sum) | LC | <0.010 | <0.010 | <0.010 | <0.010 | 0.010 |
| Terbufos-sulfone | LC | <0.010 | <0.010 | <0.010 | <0.010 | 0.010 |
| Terbufos-sulfoxide | LC | <0.010 | <0.010 | <0.010 | <0.010 | 0.010 |
| Terbumeton | GC | <0.010 | <0.010 | <0.010 | <0.010 | 0.010 |
| Terbuthylazine | GC | <0.010 | <0.010 | <0.010 | <0.010 | 0.010 |
| Terbuthylazine Desethyl | GC | <0.010 | <0.010 | <0.010 | <0.010 | 0.010 |
| Terbutryn | GC | <0.010 | <0.010 | <0.010 | <0.010 | 0.010 |
| Tetrachlorvinphos | GC | <0.010 | <0.010 | <0.010 | <0.010 | 0.010 |
| Tetraconazole | GC | <0.010 | <0.010 | <0.010 | <0.010 | 0.010 |
| Tetradifon | GC | <0.010 | <0.010 | <0.010 | <0.010 | 0.010 |
| Tetrahydrophthalimide (THPI) | GC | <0.010 | <0.010 | <0.010 | <0.010 | 0.010 |
| Tetramethrin | GC | <0.010 | <0.010 | <0.010 | <0.010 | 0.010 |
| Tetrasul | GC | <0.010 | <0.010 | <0.010 | <0.010 | 0.010 |
| TFNA | LC | <0.010 | <0.010 | <0.010 | <0.010 | 0.010 |
| TFNG | LC | <0.010 | <0.010 | <0.010 | <0.010 | 0.010 |

| | | | | | | |
|---|---|---|---|---|---|---|
| Thiabendazole | LC | <0.010 | <0.010 | <0.010 | <0.010 | 0.010 |
| Thiacloprid | LC | <0.010 | <0.010 | <0.010 | <0.010 | 0.010 |
| Thiamethoxam (SP) | LC | <0.010 | <0.010 | <0.010 | <0.010 | 0.010 |
| Thiamethoxam (sum) | LC | <0.010 | <0.010 | <0.010 | <0.010 | 0.010 |
| Thidiazuron | LC | <0.010 | <0.010 | <0.010 | <0.010 | 0.010 |
| Thifensulfuron Methyl | LC | <0.010 | <0.010 | <0.010 | <0.010 | 0.010 |
| Thiobencarb | LC | <0.010 | <0.010 | <0.010 | <0.010 | 0.010 |
| Thiocyclam | LC | <0.010 | <0.010 | <0.010 | <0.010 | 0.010 |
| Thiodicarb (SP) | LC | <0.010 | <0.010 | <0.010 | <0.010 | 0.010 |
| Thiofanox | LC | <0.010 | <0.010 | <0.010 | <0.010 | 0.010 |
| Thiofanox Sulfone | LC | <0.010 | <0.010 | <0.010 | <0.010 | 0.010 |
| Thiofanox Sulfoxide | LC | <0.010 | <0.010 | <0.010 | <0.010 | 0.010 |
| Thiometon | GC | <0.010 | <0.010 | <0.010 | <0.010 | 0.010 |
| Thiophanate-methyl (SP) | LC | <0.010 | <0.010 | <0.010 | <0.010 | 0.010 |
| Tolclofos Methyl | GC | <0.010 | <0.010 | <0.010 | <0.010 | 0.010 |
| Tolfenpyrad | LC | <0.010 | <0.010 | <0.010 | <0.010 | 0.010 |
| Tolylfluanid (SP) | LC | <0.010 | <0.010 | <0.010 | <0.010 | 0.010 |
| Tolylfluanid (Sum) | LC | <0.010 | <0.010 | <0.010 | <0.010 | 0.010 |
| Trans-Chloradane | GC | <0.01 | <0.01 | <0.01 | <0.01 | 0.01 |
| Transfluthrin | GC | <0.010 | <0.010 | <0.010 | <0.010 | 0.010 |
| Triadimefon | GC | <0.010 | <0.010 | <0.010 | <0.010 | 0.010 |
| Triadimenol | GC | <0.010 | <0.010 | <0.010 | <0.010 | 0.010 |
| Tri-Allate | GC | <0.010 | <0.010 | <0.010 | <0.010 | 0.010 |
| Triamiphos | GC | <0.010 | <0.010 | <0.010 | <0.010 | 0.010 |
| Triasulfuron | LC | <0.010 | <0.010 | <0.010 | <0.010 | 0.010 |
| Triazophos | LC | <0.010 | <0.010 | <0.010 | <0.010 | 0.010 |
| Triazoxide | LC | <0.010 | <0.010 | <0.010 | <0.010 | 0.010 |
| Trichlorfon | LC | <0.010 | <0.010 | <0.010 | <0.010 | 0.010 |
| Tricresyl phosphate | LC | <0.010 | <0.010 | <0.010 | <0.010 | 0.010 |
| Tricyclazole | LC | <0.010 | <0.010 | <0.010 | <0.010 | 0.010 |
| Tridemorph | LC | <0.010 | <0.010 | <0.010 | <0.010 | 0.010 |
| Trifloxystrobin | LC | <0.010 | <0.010 | <0.010 | <0.010 | 0.010 |
| Triflumizole (SP) | LC | <0.010 | <0.010 | <0.010 | <0.010 | 0.010 |

| | | | | | | |
|---|---|---|---|---|---|---|
| Triflumizole (Sum) | LC | <0.010 | <0.010 | <0.010 | <0.010 | 0.010 |
| Triflumizole FM 6-1 | LC | <0.010 | <0.010 | <0.010 | <0.010 | 0.010 |
| Triflumuron | LC | <0.010 | <0.010 | <0.010 | <0.010 | 0.010 |
| Trifluralin | GC | <0.010 | <0.010 | <0.010 | <0.010 | 0.010 |
| Triforine (SQ) | LC | <0.010 | <0.010 | <0.010 | <0.010 | 0.010 |
| Triticonazole | LC | <0.010 | <0.010 | <0.010 | <0.010 | 0.010 |
| Uniconazole | GC | <0.010 | <0.010 | <0.010 | <0.010 | 0.010 |
| Vamidothion | LC | <0.010 | <0.010 | <0.010 | <0.010 | 0.010 |
| Vinclozolin (SP) | GC | <0.010 | <0.010 | <0.010 | <0.010 | 0.010 |
| Zoxamide | LC | <0.010 | <0.010 | <0.010 | <0.010 | 0.010 |
| Zeta-cypermethrin | GC | <0.010 | <0.010 | <0.010 | <0.010 | 0.010 |

## Supplemental Table 7. Accuracy and Precision Summary from Blank Spike and Recovery Data[1]

| Analyte | Reference value (mg/kg) | Measured value (mg/kg) | Recovery (%) |
|---|---|---|---|
| Ag | 0.4000 | 0.412 | 103 |
| Al | 40.00 | 39.11 | 98 |
| As | 4.000 | 3.713 | 93 |
| B | 4.000 | 3.662 | 92 |
| Ba | 4.000 | 4.192 | 105 |
| Be | 4.000 | 3.919 | 98 |
| Ca | 40.00 | 42.09 | 105 |
| Cd | 0.4000 | 0.400 | 100 |
| Co | 4.000 | 3.970 | 99 |
| Cr | 4.000 | 4.100 | 103 |
| Cu | 4.000 | 4.116 | 103 |
| Fe | 40.00 | 39.48 | 99 |
| Hg[2] | 0.1000 | 0.092 | 92 |
| K | 40.00 | 53.99 | 135 |
| Li | 4.000 | 3.702 | 93 |
| Mg | 40.00 | 39.92 | 100 |
| Mn | 4.000 | 4.153 | 104 |
| Mo | 0.4000 | 0.422 | 106 |
| Na | 40.00 | 41.50 | 104 |
| Ni | 4.000 | 3.838 | 96 |
| P | 400.0 | 385.0 | 96 |
| Pb | 0.4000 | 0.413 | 103 |
| Sb | 0.4000 | 0.411 | 103 |
| Se | 4.000 | 4.151 | 104 |
| Sn | 0.4000 | 0.414 | 103 |
| Sr | 4.000 | 4.104 | 103 |
| Tl[3] | 0.4000 | 0.412 | 103 |
| U | 0.4000 | 0.362 | 90 |
| V | 4.000 | 4.121 | 103 |
| Zn | 4.000 | 4.003 | 100 |

[1] Acceptance criteria for percent limits were 75-125%. Because acceptance criteria for K was above criteria, K recovery was assessed with 4 method blanks and was found to be within method criteria (83% recovery).
[2] Result is ≤MDL
[3] Detected by the instrument, the result is >MDL but ≤MRL. Result reported considered an estimate.

## Supplemental Table 8. Method Reporting Limits for Juniper Ash Samples (n=3)

| Source of Ash | Analyte | Method Reporting Limit (mg/kg) |
|---|---|---|
| Rocky Mountain Juniper | Ag | 0.002 |
| Rocky Mountain Juniper | Al | 1.91 |
| Rocky Mountain Juniper | As | 0.008 |
| Rocky Mountain Juniper | B | 239 |
| Rocky Mountain Juniper | Ba | 3.82 |
| Rocky Mountain Juniper | Be | 0.017 |
| Rocky Mountain Juniper | Ca | 3820 |
| Rocky Mountain Juniper | Cd | 0.007 |
| Rocky Mountain Juniper | Co | 0.011 |
| Rocky Mountain Juniper | Cr | 0.143 |
| Rocky Mountain Juniper | Cu | 38.2 |
| Rocky Mountain Juniper | Fe | 153 |
| Rocky Mountain Juniper | Hg[4] | 0.009 |
| Rocky Mountain Juniper | K | 120 |
| Rocky Mountain Juniper | Li | 0.016 |
| Rocky Mountain Juniper | Mg | 382 |
| Rocky Mountain Juniper | Mn | 3.82 |
| Rocky Mountain Juniper | Mo | 0.014 |
| Rocky Mountain Juniper | Na | 2290 |
| Rocky Mountain Juniper | Ni | 0.344 |
| Rocky Mountain Juniper | P | 95.5 |
| Rocky Mountain Juniper | Pb | 0.007 |
| Rocky Mountain Juniper | Sb | 0.005 |
| Rocky Mountain Juniper | Se | 0.010 |
| Rocky Mountain Juniper | Sn | 0.006 |
| Rocky Mountain Juniper | Sr | 34.4 |
| Rocky Mountain Juniper | Tl[5] | 0.024 |
| Rocky Mountain Juniper | U | 0.007 |
| Rocky Mountain Juniper | V | 0.008 |
| Rocky Mountain Juniper | Zn | 153 |
| Eastern Red Cedar Ash | Ag | 0.002 |
| Eastern Red Cedar Ash | Al | 1.99 |
| Eastern Red Cedar Ash | As | 0.008 |
| Eastern Red Cedar Ash | B | 248 |
| Eastern Red Cedar Ash | Ba | 3.97 |
| Eastern Red Cedar Ash | Be[6] | 0.018 |
| Eastern Red Cedar Ash | Ca | 3970 |

[4] Result is ≤MDL

[5] Detected by the instrument, the result is >MDL but ≤MRL. Result reported considered an estimate.

[6] Detected by the instrument, the result is >MDL but ≤MRL. Result reported considered an estimate.

| | | |
|---|---|---|
| Eastern Red Cedar Ash | Cd | 0.007 |
| Eastern Red Cedar Ash | Co | 0.012 |
| Eastern Red Cedar Ash | Cr | 0.149 |
| Eastern Red Cedar Ash | Cu | 39.7 |
| Eastern Red Cedar Ash | Fe | 159 |
| Eastern Red Cedar Ash | Hg[7] | 0.009 |
| Eastern Red Cedar Ash | K | 125 |
| Eastern Red Cedar Ash | Li | 0.017 |
| Eastern Red Cedar Ash | Mg | 397 |
| Eastern Red Cedar Ash | Mn | 3.97 |
| Eastern Red Cedar Ash | Mo | 0.015 |
| Eastern Red Cedar Ash | Na | 11.9 |
| Eastern Red Cedar Ash | Ni | 0.358 |
| Eastern Red Cedar Ash | P | 99.3 |
| Eastern Red Cedar Ash | Pb | 0.007 |
| Eastern Red Cedar Ash | Sb | 0.005 |
| Eastern Red Cedar Ash | Se | 0.010 |
| Eastern Red Cedar Ash | Sn | 0.006 |
| Eastern Red Cedar Ash | Sr | 35.8 |
| Eastern Red Cedar Ash | Tl[8] | 0.025 |
| Eastern Red Cedar Ash | U | 0.007 |
| Eastern Red Cedar Ash | V | 0.008 |
| Eastern Red Cedar Ash | Zn | 159 |
| Vendor-purchased Ash | Ag | 0.002 |
| Vendor-purchased Ash | Al | 386 |
| Vendor-purchased Ash | As | 0.008 |
| Vendor-purchased Ash | B | 24.1 |
| Vendor-purchased Ash | Ba | 3.86 |
| Vendor-purchased Ash | Be | 0.017 |
| Vendor-purchased Ash | Ca | 3860 |
| Vendor-purchased Ash | Cd | 0.007 |
| Vendor-purchased Ash | Co | 0.012 |
| Vendor-purchased Ash | Cr | 0.145 |
| Vendor-purchased Ash | Cu | 38.6 |
| Vendor-purchased Ash | Fe | 154 |
| Vendor-purchased Ash | Hg[9] | 0.009 |
| Vendor-purchased Ash | K | 122 |
| Vendor-purchased Ash | Li | 0.016 |
| Vendor-purchased Ash | Mg | 386 |
| Vendor-purchased Ash | Mn | 3.86 |
| Vendor-purchased Ash | Mo | 2.89 |
| Vendor-purchased Ash | Na | 11.6 |

[7] Result is ≤MDL
[8] Detected by the instrument, the result is >MDL but ≤MRL. Result reported considered an estimate.
[9] Detected by the instrument, the result is >MDL but ≤MRL. Result reported considered an estimate.

| | | |
|---|---|---|
| Vendor-purchased Ash | Ni | 0.347 |
| Vendor-purchased Ash | P | 96.5 |
| Vendor-purchased Ash | Pb | 1.39 |
| Vendor-purchased Ash | Sb | 0.005 |
| Vendor-purchased Ash | Se | 0.010 |
| Vendor-purchased Ash | Sn | 0.006 |
| Vendor-purchased Ash | Sr | 34.7 |
| Vendor-purchased Ash | Tl | 0.024 |
| Vendor-purchased Ash | U | 0.007 |
| Vendor-purchased Ash | V | 0.008 |
| Vendor-purchased Ash | Zn | 154 |
